# Effect of a model consultation informed by guidelines on recorded quality of care of osteoarthritis (MOSAICS): a cluster randomised controlled trial in primary care

**DOI:** 10.1016/j.joca.2017.05.017

**Published:** 2017-10

**Authors:** K.P. Jordan, J.J. Edwards, M. Porcheret, E.L. Healey, C. Jinks, J. Bedson, K. Clarkson, E.M. Hay, K.S. Dziedzic

**Affiliations:** † Arthritis Research UK Primary Care Centre, Research Institute for Primary Care & Health Sciences, David Weatherall Building, Keele University, Staffordshire, ST5 5BG, UK; ‡ Keele Clinical Trials Unit, David Weatherall Building, Keele University, Staffordshire, ST5 5BG, UK

**Keywords:** Osteoarthritis, General practice, Implementation, Primary care, Guidelines

## Abstract

**Objective:**

To determine the effect of a model osteoarthritis (OA) consultation (MOAC) informed by National Institute for Health and Care Excellence (NICE) recommendations compared with usual care on recorded quality of care of clinical OA in general practice.

**Design:**

Two-arm cluster randomised controlled trial.

**Setting:**

Eight general practices in Cheshire, Shropshire, or Staffordshire UK.

**Participants:**

General practitioners and nurses with patients consulting with clinical OA.

**Intervention:**

Following six-month baseline period practices were randomised to intervention (*n* = 4) or usual care (*n* = 4). Intervention practices delivered MOAC (enhanced initial GP consultation, nurse-led clinic, OA guidebook) to patients aged ≥45 years consulting with clinical OA. An electronic (e-)template for consultations was used in all practices to record OA quality care indicators.

**Outcomes:**

Quality of OA care over six months recorded in the medical record.

**Results:**

1851 patients consulted in baseline period (1015 intervention; 836 control); 1960 consulted following randomisation (1118 intervention; 842 control). At baseline wide variations in quality of care were noted. Post-randomisation increases were found for written advice on OA (4–28%), exercise (4–22%) and weight loss (1–15%) in intervention practices but not controls (1–3%). Intervention practices were more likely to refer to physiotherapy (10% vs 2%, odds ratio 5.30; 95% CI 2.11, 13.34), and prescribe paracetamol (22% vs 14%, 1.74; 95% CI 1.27, 2.38).

**Conclusions:**

The intervention did not improve all aspects of care but increased core NICE recommendations of written advice on OA, exercise and weight management. There remains a need to reduce variation and uniformly enhance improvement in recorded OA care.

**Trial registration number:**

ISRCTN06984617.

## Introduction

Osteoarthritis (OA) is a major cause of pain and disability worldwide^1^, ^2^. Most patients with clinical OA are seen and managed in primary care, and the UK National Institute for Health and Care Excellence (NICE) has identified a set of core interventions which can be offered to all patients consulting with OA in primary care. Yet much primary care for OA patients in the UK does not adhere to NICE guidance, including the core items of education and information provision, and advice and referral for exercise and weight management^1^, ^3^, ^4^, ^5^, ^6^. Internationally, the situation is similar^7^, ^8^ and a change in models of care for OA has been proposed^9^.

A systematic review has previously identified some limited evidence to support primary care collaborative care models and multidisciplinary case management as complex interventions to improve OA care^10^. Strategies to improve quality of primary care for long-term conditions in the UK have included use of computerised templates and decision support systems^11^, health trainers^12^, promotion of self-management^13^, and educational intervention^14^. Although some risk factors for OA are addressed by the health trainer model (weight management, exercise/physical activity), there have been few successful attempts to enhance OA care in general practice.

The MOSAICS (Managing OSteoArthritis In ConsultationS) study was a cluster randomised controlled trial to test a complex patient-focused intervention, namely a model OA consultation during which the core NICE OA recommendations are delivered. This was developed using the Whole Systems Informing Self-Management Engagement (WISE) model^15^ and incorporated an OA Guidebook developed with user involvement, an enhanced OA consultation, and access to a practice based nurse-led OA clinic^16^, ^17^. The MOSAICS study aimed to assess:

• The effectiveness of the intervention on the quality of primary care for patients aged ≥45 years consulting with clinical OA.
• The impact, feasibility and acceptability of the model OA consultation in primary care.

We report here the practice-level results addressing the study question of whether the intervention (model OA consultation) increases the uptake of NICE OA recommendations by general practices taking part in MOSAICS, as measured by quality indicators of OA care in the practices' electronic health records (EHR). A quality indicator was defined as “*a measurable element of practice performance for which there is evidence or consensus that it can be used to assess the quality, and hence change in the quality, of care provided*”^18^. We also report on adverse events.

## Methods

### Study design

MOSAICS was a mixed methods study with a two arm cluster randomised controlled trial conducted in eight general practices in Cheshire, Shropshire, or Staffordshire, UK. The protocol has been published^17^ and the patient-level self-reported outcomes for clinical effectiveness will be reported elsewhere.

The MOSAICS study has two key parts: a population survey that took place between May 2011 and April 2012 and a cluster randomised trial that was conducted from May 2012 to February 2014 by the Arthritis Research UK Primary Care Centre, Keele University, UK. The study was approved by the North West 1 Research Ethics Committee, Cheshire (REC reference: 10/H1017/76) and monitored by an Independent Trial Steering Committee and Data Monitoring Committee.

Cluster randomisation at the practice level was used to prevent contamination by clinicians as it was expected GPs would be unable to manage patients allocated to the control arm differently to those allocated to the intervention arm. It may also better develop a community of practice for OA care within a cluster. The evaluation of the intervention used anonymised medical records to allow the analysis of the management and care of a large number of patients without recruitment bias and the attrition and non-consent issues of self-reported patient evaluation. By using medical record information for measuring the outcomes, all eligible patients in the practices were included.

### Participants

Practices which were members of the Central England Primary Care Research Network or a Keele Research Network Practice, and used the EMIS computerised system were approached sequentially until eight agreed to take part. Ten general practices were invited to participate. Reasons for non-participation were recent engagement with teaching medical students and involvement with other research^19^.

All health care professionals (general practitioners and practice nurses) from the eight randomised practices and their respective practice populations aged ≥45 years consulting with clinical OA (diagnosed OA or recorded peripheral joint pain) formed the sampling frame for the cluster trial.

During a six month baseline period prior to randomisation, all practices received a resource pack of written advice for patients, with examples of OA leaflets provided by Arthritis Research UK, Arthritis Care and NICE. Training of health care professionals in the trial intervention occurred after randomisation.

Patients eligible for inclusion were aged ≥45 years and had at least one consultation recorded as clinical OA defined as an OA diagnostic Read code or a code for joint pain (hand/wrist, hip, knee, foot/ankle) during the study period. In UK primary care, morbidities are generally entered using Read Codes, a hierarchical coding system structured into chapters. For example, codes under Chapter N represent ‘Musculoskeletal and Connective Tissue Diseases’. GPs may often enter symptom codes rather than diagnosis codes and using only OA diagnostic Read codes means patients presenting with OA symptoms will be missed^20^, ^21^. Joint pain codes likely to represent OA had previously been determined by six academic general practitioners with an interest in musculoskeletal conditions^22^. The current analysis was performed on the anonymised EHR data of all patients fulfilling the eligibility criteria.

### Randomisation

Following the six month baseline period, practices were randomised into intervention (model OA consultation, four practices) or to continue with usual care (four practices). Practices were randomly allocated, stratified by practice list size, by administrative staff at the Keele Clinical Trials Unit who had no clinical involvement in the trial. The trial statisticians were kept blind to the allocation until after the analysis.

## Intervention

### The model OA consultation

The development of the intervention has been published elsewhere^17^, ^23^, ^24^. Briefly, using the findings of two consensus exercises^23^, ^25^ and theoretical models to guide self-management^26^ and support patient behaviour change^27^, ^28^, a model OA consultation was developed. This comprised an enhanced initial consultation with the GP and provision of a nurse-led OA clinic, both supported by use of an OA Guidebook, and was delivered to patients aged ≥45 years presenting with clinical OA (Appendix 1).

### Training

Training and educational packages were developed by drawing on Michie *et al.*^28^, ^29^. Intervention practices received practice updates on core NICE recommendations for OA (diagnosis; written information [the OA guidebook], exercise and physical activity, healthy eating, pain management). GPs received training on how to deliver the initial consultation for new or established OA patients during four sessions (2 h ×3, 1 h ×1) utilizing simulated patients in skills training sessions^16^. The procedure for referring to a practice nurse for a follow-up OA consultation was discussed. Practice nurses received four days of training on how to support and enable patients to self-manage OA, using a patient-centred approach, the OA guidebook, goal setting, pain management (analgesia and exercise) and the core NICE recommendations (information and advice, strengthening exercise and aerobic fitness training, and weight management)^30^.

Control practices received no training, guidebook or OA nurse clinic, and continued usual care alongside the resource pack of written advice for patients given in the pre-randomisation baseline period.

### Outcomes

The outcomes were the recorded achievement (achieved vs not achieved) of fourteen quality indicators of care for patients presenting with clinical OA during the six month period after randomisation and training. This was assessed through the use of quality indicators derived from a systematic review^31^ with additional measures derived from the NICE OA guidelines (Box 1)^1^. They cover four domains: assessment (pain, function, body mass index (BMI), X-ray use), core management (OA information, exercise advice, weight loss advice), other non-pharmacological management (physiotherapy referral), and pharmacological management (paracetamol, topical non-steroidal anti-inflammatory drugs (NSAIDs), gastroprotection). For the core management indicators, indicator achievement was defined as the information being given verbally, written, or deemed by the clinician as not appropriate. However, we also assessed whether there had been increases in the level of written information and advice as this is the core NICE recommendation^1^.

Box 1 **Quality indicators of primary care of osteoarthritis**

**Table:** 

Domain	Quality indicator	Indicator source∗	Data source	Evidence of achievement	Change signalling care improved†
Assessment	Pain assessed	Review	e-Template	Recorded level of pain‡	Increase
	Function assessed	Review	e-Template	Recorded level of function‡	Increase
	BMI measurement/weight record	Review	e-Template & routine EHR	Recorded BMI or weight	Increase
	X-ray requested	Guideline	Routine EHR	Recorded X-ray of knee, hip, hand, or foot	Decrease
Core interventions	OA information	Review	e-Template	Recorded as verbal or written; or not appropriate∗∗	Increase
	Written OA information	Guideline	e-Template	Recorded as written	Increase
	Exercise advice	Review	E-Template	Recorded as verbal or written; or not necessary or not appropriate∗∗	Increase
	Written exercise advice	Guideline	e-Template	Recorded as written	Increase
	Weight loss advice††	Review	e-Template	Recorded as verbal or written; or not appropriate∗∗	Increase
	Written weight loss advice††	Guideline	e-Template	Recorded as written	Increase
Non-pharmacological interventions	Consideration of physiotherapy referral	Guideline	e-Template	Recorded as offered; or not necessary or not appropriate∗∗	Increase
	Physiotherapy referral made	Guideline	Routine EHR	Recorded referral to physiotherapy	Increase
Pharmacological interventions	Consideration of paracetamol use	Review	e-Template	Recorded as tried, offered, or declined full dose; or not appropriate‡‡	Increase
	Paracetamol prescribed	Review	Routine EHR	Recorded prescription	Increase
	Consideration of topical NSAID use	Guideline	e-Template	Recorded as tried, offered or declined full dose; or not appropriate‡‡	Increase
	Topical NSAID prescribed	Guideline	Routine EHR	Recorded prescription	Increase
	Gastroprotection (PPI use with oral NSAIDs)	Review	Routine EHR	Recorded prescription (if oral NSAID prescribed)	Increase

PPI = proton pump inhibitor. Clinicians were asked to record “not appropriate” when they considered a patient not eligible for a process of care.

∗ Systematic review^31^ or NICE guideline^1^, indicators taken from routine record had to be within 14 days of a clinical OA consultation.

† Compared to control group.

‡ None, mild, moderate, severe.

∗∗ Not this time or no entry indicates non-achievement.

†† In those with recorded BMI ≥ 25 in previous 3 years.

‡‡ Unknown or no entry indicates non-achievement.

Alt-text: Box 1

Recorded achievement of quality indicators was identified via two sources: information routinely entered in the EHR as part of standard care and that entered through an electronic template (“e-template”) developed to allow clinicians to complete and capture information not routinely recorded (Box 1). The e-template was installed in all practices at the start of the six-month baseline period and was automatically triggered at any consultation with an entry of the same Read codes used to identify patients for the trial. Clinicians could choose to complete all, some, or none of the e-template. As previously reported, the e-template was found to be associated with an increased recording of weight and prescription of NICE-recommended first-line analgesics (paracetamol, topical NSAIDS) in the baseline period but other recorded care remained stable^32^.

Quality indicators could be achieved at the first consultation for clinical OA within the trial period or the following 120 days (to allow time for the patient to see the practice nurse). For indicators assessed through the routine record, they also had to be recorded within 14 days of a recorded consultation for clinical OA.

The percentage of patients in the intervention practices with a recorded practice nurse consultation (as directed in the model OA consultation) were identified from medical records as a measure of treatment fidelity.

### Adverse events

Adverse events that may be related to the content of the model OA consultation and quality of care indicators were selected based on the NICE 2008 OA guidelines^1^ and recommendations of the Trial Steering Committee, and identified in the EHR from date of first OA consultation during the trial period up the last point of record download (31/8/2013).

### Sample size

Sample size for the trial was based on the clinical effectiveness component^17^. A priori, based on a 10% annual consultation prevalence for clinical OA in those aged ≥45^22^, and a population base of 30,000 adults aged ≥45 years across the eight general practices, we estimated there would be 3000 patients consulting annually for clinical OA.

### Statistical analysis

The analysis compared the intervention and control practices on recorded achievement of the individual quality indicators of care in patients consulting with clinical OA during the trial period (six months after randomisation and training). We determined practice-specific baseline levels of recorded quality indicator achievement. Baseline was taken as the first six months the e-template was introduced in the practices (prior to randomisation and training) and was based on patients with a recorded OA or joint pain code during that period. During the six month trial period, we identified the initial clinician recorded as seen by each patient for clinical OA in that period.

Multilevel logistic regression models (patients nested within initial clinician seen) were used to determine differences between intervention and control practices during the trial period in the achievement of each quality indicator. The models were adjusted for age, gender, whether the initial consultation was recorded as diagnosed OA or given a joint pain code, and baseline level of quality indicator achievement of the patient's practice. Results are presented as odds ratios (OR) with 95% CI.

Sensitivity analyses restricted the analysis to: (1) patients with at least one recorded entry on the e-template, (2) new consulters (defined as first clinical OA consultation since introduction of the e-template and with at least 365 days since any clinical OA consultation), (3) patients with a recorded diagnosis of OA.

To assess the likely effect of treatment fidelity, we descriptively compared recorded achievement of quality indicators, in the intervention practices only, between patients with a record of attendance at a practice nurse clinic and those without.

Differences in adverse events were analysed using chi-squared tests or Fisher's Exact Test as appropriate. Stata/MP 13.1, MLwiN v2.29 and the Stata command ‘runmlwin’ were used for the analyses^33^, ^34^.

## Results

**Fig. 1:**
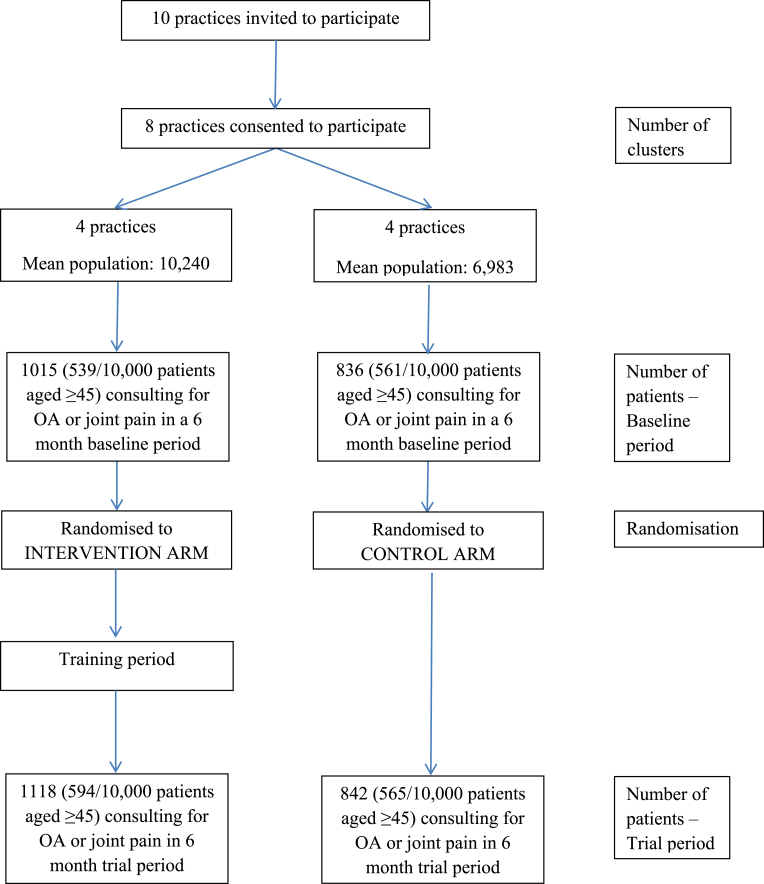
Flowchart of practices and patients included in study.

Mean registered populations for the practices were 10,240.5 (intervention) and 6983.3 (control). There were 1118 patients recorded with clinical OA during the six month trial period in the intervention practices, and 842 patients in the control practices (Fig. 1). Mean age of patients was 66.2 years (SD 12.34, intervention) and 66.5 (SD 11.93, control). 59% were female in the intervention practices, 61% in the control practices. The e-template fired for 1061 (95%) of the 1118 patients in the intervention practices and 757 (90%) of the 842 patients in the control practices. The reason for the template failing to fire for the remaining patients is unknown.

41% (baseline) and 45% (trial period) of patients in the intervention practices received an OA diagnosis rather than a joint pain code compared to 23% and 29% in the control practices, respectively. During the trial period, there were 63 clinicians who first saw a patient in the intervention practices (seeing a median of 10 patients; IQR 2, 29) and 50 clinicians in the control practices (median 11 patients; IQR 2, 26).

### Recorded achievement of quality indicators

There was wide variation in recorded achievement of the quality indicators during the baseline pre-randomisation period, measured through the e-template, between clinicians and between practices. For example, as previously reported^32^, in clinicians seeing more than the median number of clinical OA patients, a quarter failed to achieve any e-template measured indicator for more than half of their patients but another quarter achieved at least one indicator for more than 88% of their patients. This variation was reflected in wide baseline differences between the trial arms and there was a fall in recorded achievement of e-template measured indicators between baseline and trial period for both intervention and control practices, although this was not apparent in patients who had at least one entry on the e-template.

There were no statistically significant differences between intervention and control practices in the recorded achievement of the assessment quality indicators although X-ray requests reduced in the intervention arm (25–15%) but increased in the control practices (3–6%, OR 0.45; 95% CI 0.12, 1.72, Table I). There were also no statistically significant differences in the general indicators of core management. However, a record of the health care professional supplying written information on OA increased in the intervention practices from 4% of patients in the baseline period to 28% in the trial period and remained stable in the control practices (1–2%, OR 23.60, 95% CI 7.39, 75.40, Table II). Written exercise advice and written weight loss advice in those overweight also increased significantly in the intervention practices in comparison to the control practices.

**Table I:** Comparison between intervention and control arms in recorded quality indicator achievement

Domain		Baseline period		Trial period		OR† (95% CI)	ICC‡
		Intervention *n*∗ (%)	Control *n*∗ (%)	Intervention *n*∗ (%)	Control *n*∗ (%)		
	No. of consulters∗∗	1015/981	836/749	1118/1061	842/757		
Assessment	Pain assessment	707 (72)	390 (52)	617 (58)	318 (42)	1.35 (0.58, 3.14)	0.36
	Function assessment	691 (70)	384 (51)	611 (58)	309 (41)	1.15 (0.49, 2.71)	0.35
	Weight record	278 (27)	154 (18)	309 (28)	144 (17)	1.36 (0.80, 2.33)	0.20
	X-ray requested	250 (25)	22 (3)	163 (15)	47 (6)	0.45 (0.12, 1.72)	0.22
Core management	Information given	578 (59)	274 (37)	554 (52)	268 (35)	1.34 (0.61, 2.96)	0.34
	Exercise advice	582 (59)	285 (38)	526 (50)	246 (32)	1.53 (0.75, 3.13)	0.28
	Weight loss advice††	325 (53)	159 (34)	341 (49)	136 (31)	1.24 (0.61, 2.52)	0.28
Non-pharmacological management	Physiotherapy referral considered	426 (43)	192 (26)	348 (33)	173 (23)	1.45 (0.61, 3.40)	0.29
	Physiotherapy referral made	90 (9)	35 (4)	111 (10)	19 (2)	5.30 (2.11, 13.34)	0.20
Pharmacological management	Paracetamol considered	625 (64)	349 (47)	554 (52)	284 (38)	1.42 (0.71, 2.85)	0.29
	Paracetamol prescribed	164 (16)	155 (19)	241 (22)	117 (14)	1.74 (1.27, 2.38)	0.03
	Topical NSAID considered	540 (55)	295 (39)	501 (47)	275 (36)	0.97 (0.48, 1.95)	0.28
	Topical NSAID prescribed	267 (26)	194 (23)	327 (29)	186 (22)	1.21 (0.83, 1.76)	0.09
	PPI prescribed‡‡	63 (35)	27 (23)	69 (39)	50 (36)	0.92 (0.43, 1.98)	0.14

∗ Number of patients with record of achievement of indicator.

† Adjusted for age, gender, coded OA or joint pain, practice level of achievement in baseline period and accounting for clustering by clinician, reference is control group.

‡ Estimated intraclass correlation coefficient based on adjusted model.

∗∗ Number consulting for clinical OA in trial period and hence with routine record information/number for whom e-template fired.

†† In those recorded as overweight: baseline period intervention *n* = 615, control *n* = 470; trial period intervention *n* = 698, control *n* = 439.

‡‡ On date of NSAID prescription in those prescribed oral NSAIDs: baseline period intervention *n* = 181, control *n* = 119; trial period intervention *n* = 176, control *n* = 137. PPI = proton pump inhibitor.

**Table II:** Recorded achievement of quality indicators based on core NICE written recommendations by trial arm

	Baseline period		Trial period		OR∗ (95% CI)
	Intervention	Control	Intervention	Control	
	*n* (%)	*n* (%)	*n* (%)	*n* (%)	
All consulters firing e-template					
No. of consulters	981	749	1061	757	
Written information	36 (4)	6 (0.8)	296 (28)	12 (2)	23.60 (7.39, 75.40)
Written exercise advice	38 (4)	8 (1)	232 (22)	7 (0.9)	21.49 (6.62, 69.72)
Written weight loss advice†	7 (1)	1 (0.2)	104 (15)	2 (0.5)	27.94 (3.56, 219.17)
Coded with osteoarthritis diagnosis					
No. of consulters	410	178	483	218	
Written information	23 (6)	2 (1)	201 (42)	7 (3)	26.92 (6.33, 114.51)
Written exercise advice	24 (6)	2 (1)	158 (33)	2 (0.9)	40.49 (5.64, 290.56)
Written weight loss advice‡	7 (3)	1 (0.9)	79 (24)	1 (0.8)	∗∗

∗ Adjusted for age, gender, coded OA or joint pain, practice level of achievement in baseline period and accounting for clustering by clinician, reference is control group.

† In those recorded as overweight: baseline period intervention *n* = 615, control *n* = 470; trial period intervention *n* = 698, control *n* = 439.

‡ In those recorded as overweight: baseline period intervention *n* = 272, control *n* = 114; trial period intervention *n* = 335, control *n* = 132.

∗∗ Model failed to converge.

Physiotherapy referral remained stable in intervention practices (9% baseline, 10% trial period) and decreased slightly in control practices (4–2%; comparison in trial period between intervention practices and control practices: OR 5.30; 95% CI 2.11, 13.34). Prescribing of paracetamol increased from the baseline period in the intervention arm (16–22%) and decreased in the control arm (19–14%, OR 1.74; 95% CI 1.27, 2.38).

Restricting the analysis of indicators measured through the e-template to patients with at least one entry suggested a higher rate in the intervention practices of consideration of paracetamol use (OR 2.01; 95% CI 0.91, 4.41) and advice to exercise (OR 1.88; 95% CI 0.93, 3.79), albeit not statistically significant (Appendix Table I). As in the main analysis, there were decreases from baseline in recorded achievement of the indicators measured through the e-template in new consulters and just those with an OA diagnosis. Restricting the analyses of indicators recorded through the routine records to new consulters for clinical OA did not change the findings from the main analysis (Appendix Table II). In those with an OA diagnostic code only, patients in the intervention practices were additionally more likely to have their weight recorded (OR 3.07; 95% CI 1.37, 6.90) than those in the control practices (Appendix Table III). There were larger increases in the intervention arm in those with an OA diagnosis for the core written aspects of management (written information 6–42%; written exercise advice 6–33%; written weight loss advice 3–24%) than seen in the main analysis (Table II).

220 (21%) of patients with clinical OA in the intervention practices had a record of attending a practice nurse clinic. There was a higher percentage of patients with an OA diagnosis in those attending the nurse clinic than in those who did not attend the nurse clinic (68% vs 40%). Except for physiotherapy referral and X-ray request, those who saw a practice nurse had higher levels of recorded achievement in indicators measured either through the routine records or through the e-template. In particular, 89% of those consulting a practice nurse received written information compared to 24% of those who did not (in those who had at least one entry on the e-template). There were also higher levels of written exercise advice (80% vs 13%) and written weight loss advice (44% vs 10%) (Table III).

**Table III:** Recorded quality indicator achievement in those attending nurse clinics and those who did not during trial period – intervention arm only (4 practices)

		Did not attend nurse clinic		Attended nurse clinic
		All *n*∗ (%)	≥1 e-template entry *n*∗ (%)	*n*∗ (%)
	No. of consulters†	840	416	220
Assessment	Pain assessment	398 (47)	398 (96)	218 (99)
	Function assessment	392 (47)	392 (94)	218 (99)
	Weight record	136 (16)	N/A	168 (76)
	X-ray requested	118 (14)	N/A	36 (16)
Core management	Information given	338 (40)	338 (81)	215 (98)
	Written information	100 (12)	100 (24)	195 (89)
	Exercise advice	309 (37)	309 (74)	216 (98)
	Written exercise advice	55 (7)	55 (13)	177 (80)
	Weight loss advice‡	193 (37)	193 (69)	147 (87)
	Written weight loss advice‡	29 (5)	29 (10)	75 (44)
Non-pharmacological management	Physiotherapy referral considered	215 (26)	215 (52)	132 (60)
	Physiotherapy referral made	91 (11)	N/A	18 (8)
Pharmacological management	Paracetamol considered	352 (42)	352 (85)	201 (91)
	Paracetamol prescribed	160 (19)	N/A	76 (35)
	Topical NSAID considered	316 (38)	316 (76)	184 (84)
	Topical NSAID prescribed	219 (26)	N/A	94 (43)
	PPI∗∗ prescribed	54 (38)	N/A	14 (45)

∗ Number (%) of patients with record of achievement of indicator.

† One patient excluded as recorded nurse clinic was before start of analysis period.

‡ In those recorded as overweight: not attended nurse clinic *n* = 528, not attended nurse clinic but at least 1 e-template entry *n* = 279, attended nurse clinic *n* = 169.

∗∗ In those prescribed NSAID, not attended nurse clinic *n* = 142, attended nurse clinic = 32. PPI = proton pump inhibitor. N/A = not applicable as quality achievement assessed using routine records.

### Adverse events

An adverse event was recorded in 13% of patients in the intervention arm and 11% in the control arm (Table IV). Differences between arms were small and the one significant difference between arms was for heart failure (1.5% intervention arm vs 0.5% control arm). Of note, only two of the 17 patients with heart failure in the intervention arm had been prescribed either paracetamol or an oral NSAID for clinical OA during the trial period.

**Table IV:** Comparison between intervention and control arms on adverse events recorded from first consultation for OA or joint pain in trial period to thirty first August 2013

		Intervention	Control	*P-*value∗
No. of consulters		1118	842	
No. of days of follow-up Median (IQR)		416 (360, 460)	408 (355, 451)	
Death	*n* (%)	1 (0.1)	1 (0.1)	0.68
Heart failure	*n* (%)	17 (1.5)	4 (0.5)	0.03††
New heart failure†	*n* (%)	9 (0.8)	0 (0)	0.006††
Gastrointestinal	*n* (%)	9 (0.8)	9 (1.1)	0.54
Renal impairment†	*n* (%)	19 (1.7)	11 (1.3)	0.48
Liver impairment/failure	*n* (%)	0 (0)	0 (0)	–
Hypersensitivity‡	*n* (%)	1 (0.1)	2 (0.2)	0.40
Asthma flare†	*n* (%)	19 (1.7)	20 (2.4)	0.29
Renal failure∗∗	*n* (%)	2 (0.2)	0 (0)	0.33
Myocardial infarction	*n* (%)	2 (0.2)	5 (0.6)	0.13
Stroke	*n* (%)	14 (1.3)	5 (0.6)	0.14
New stroke†	*n* (%)	8 (0.7)	2 (0.2)	0.12
Fall	*n* (%)	65 (5.8)	39 (4.6)	0.25
Infection	*n* (%)	6 (0.5)	1 (0.1)	0.12
Deep vein thrombosis	*n* (%)	0 (0)	0 (0)	–
Leg amputation	*n* (%)	0 (0)	1 (0.1)	0.43
Septic arthritis	*n* (%)	0 (0)	0 (0)	–
Any adverse event	*n* (%)	146 (13.1)	96 (11.4)	0.27

∗ Chi-squared Test or Fisher's Exact Test as appropriate.

† New cases only, no record in 2 years prior to index date.

‡ Includes angioedema and new cases of wheeze (no record in 2 years prior to index date).

∗∗ Acute renal failure or chronic renal failure with no record of renal failure in 2 years prior to index date.

†† *P* < 0.05. Index date = date of first consultation for OA or joint pain in trial period.

## Discussion

A model OA consultation, informed by NICE recommendations and incorporating an enhanced initial GP consultation, nurse-led OA clinic, and OA Guidebook, compared with usual care, substantially increased uptake of core written non-pharmacological recommendations, though there remained scope for further improvement. The model OA consultation produced higher levels of prescribing of simple analgesia (paracetamol) and physiotherapy referral. There was a reduction in referral for X-ray in the intervention practices (although not statistically significant) and little evidence that the model OA consultation was associated with a higher number of adverse events. However, wide variation in recorded management was identified and evidence of improvement in recorded achievement was not consistent across all indicators.

A novelty of our study was use of anonymised practice-level data to study the effect of the intervention on all patients consulting with OA or joint pain. Uptake of recommended NICE management of OA were measured using previously identified quality indicators of OA care^31^ captured via an e-template, and routinely recorded information. To enhance the uptake of NICE OA recommendations we used theory-derived interventions, clinical champions, outreach visits, theory-informed training, funded practice nurse clinics, supply of high quality patient information, and a model OA consultation to deliver evidence-based recommendations. The extent to which each of these approaches contributed independently cannot be determined. The model OA consultation had a strong theoretical underpinning using the WISE model to define self-management and patient information and the Theoretical Domains Framework to develop training to deliver the consultation^13^, ^16^.

Our earlier work had shown that the template was a feasible way for GPs to record care, and that the introduction of the template alone had positive effects on quality care such as prescribing^32^. Introducing the model OA consultation had no discernible additional influence on the level of recording of items on the e-template beyond baseline. However, despite the limited number of practices in the trial and wide variation across practices, there was an important and statistically significant improvement in a key component of NICE guidance, namely the provision of written information about OA and written advice about exercise and weight control, in the intervention compared with control practices.

A strength was introduction of the e-template and familiarisation six months prior to randomisation to capture for the first time information on recommended indicators of quality of care not routinely captured in the EHR. The e-template alone increased the use of topical NSAIDS^32^ which reduced the likelihood of detecting further increases as a result of the model OA consultation. Use of paracetamol also showed a trend in favour of increased use in the baseline period, however there was a further statistically significant increase in use following implementation of the model OA consultation. The template failed to fire for a small group of patients. Whilst the reason for this is unknown, it is unlikely to have introduced any bias. The baseline level of achievement in various domains pre-randomisation was already high when compared with other published estimates of recorded quality of care^4^, ^5^, ^7^, ^8^. Levels of achievement of OA quality indicators as measured through the e-template fell generally from baseline levels, possibly due to initiative fatigue in use of the template. On completion of the research however seven of the eight practices chose to continue with the e-template. We also, in a sensitivity analysis, restricted analysis to patients with at least one e-template entry to try and overcome some of the influence of the fall in overall recording. However, the higher baseline levels of quality achievement compared to previous estimates, general fall in recording, and baseline variation between practices and health care professionals limited investigation of the potential effect of the intervention. There was an imbalance in the number of patients between arms due to the inclusion of one much larger practice. We included patients with consultations coded as knee, hip, hand/wrist and foot/ankle pain, as non-specific pain at these sites in older adults is most likely to be underlying OA. Recorded joint pain in other sites which may present as OA (shoulder and elbow) were not included, however these sites made up just 2% of OA diagnosed consultations during the trial period. In the analysis, we clustered patients within clinicians rather than practices. We performed a sensitivity analysis (data not shown) with practice as an extra level in the multilevel models. This showed the majority of variation was at clinician level and did not change the findings. The extent to which the recorded quality of care reflects the actual delivery of care is not known. Given quality of care is necessarily measured across several indicators, the testing of multiple comparisons could not be avoided and increased the possibility that identified differences between arms were due to chance.

Only 21% of patients in the intervention arm attended the practice nurse clinics. Referral by the GP and attendance by the patient were optional. Patients with an OA diagnosis were more likely to attend and had increased uptake of core treatments suggesting that making a formal OA diagnosis was linked to management. It is possible those given an OA diagnosis have more severe pain or functional limitation although other work suggests that known risk factors (older age, obesity) are more strongly linked to OA diagnosis than severity^20^.

The provision of OA guidebooks in the intervention arm was captured by the increased uptake of written information on OA. This is an important outcome for the trial given the recent NICE Quality Standards for OA which highlights the importance of providing written information about OA and its management^35^. Access to weight loss advice and support is recommended in the NICE guidance and is regarded as a care quality indicator^31^. The increased use of written weight loss advice is another strength of the intervention. Previous studies have shown reliance by GPs on pharmacological management of OA^3^, so the increase in these non-pharmacological core interventions is encouraging. Further work is required to understand the extent to which provision of written information and advice affects patient outcomes. X-ray use declined in the intervention arm which is in line with NICE recommendations.

There was an increased incidence of heart failure in the intervention arm. As only two of the 17 patients with heart failure had been prescribed paracetamol or oral NSAIDs, it seems unlikely that this is due to a pharmacological effect, and it seems clinically implausible that the noted statistically significant difference is caused by the intervention.

In our novel practice-level analysis of anonymised data from all consulters with OA and joint pain, we have shown that a model OA consultation intervention which provides additional resources for a primary care-based OA service, notably a patient guidebook and practice nurse referral clinics, did not lead to improvements on all indicators of quality of OA care. However there was improved achievement of NICE guidance targets for written information and advice, and some small but additional beneficial effects on prescribing and referrals.

### Patient and public involvement (PPI)

The Arthritis Research UK Primary Care Centre at Keele University is committed to taking an explicit and systematic approach to involving patients and the public in research. For this trial, a Research Users Group worked in collaboration with researchers on a wide range of tasks including: development and design of the OA guidebook^24^, developing training for GPs and practice nurses, grant co-applicant and Steering Committee Membership.

## Author contributions

KPJ was involved in design of the study, wrote the analysis plan, cleaned the data, led the analysis and drafted and revised the paper; JJE was involved in design of the study, led development of the outcome measures, contributed to analysis and drafted and revised the paper; MP led development of the intervention, contributed to design of the study and revised the paper; ELH contributed to development of the intervention and design of the study, and revised the paper; CJ, JB, EMH all contributed to design of the study and revised the paper; KC coordinated the study and revised the paper; KSD is PI for the study and led the design of the study and development of intervention, and drafted and revised the paper. All authors have approved the final version.

## Conflicts of interests

KSD was a member of the NICE Osteoarthritis Guidelines Development Group CG 59 (2008) and CG 177 (2014) and a member of the NICE Quality Standards Group for Osteoarthritis. KSD has been an invited speaker at Bone and Joint Decade 2015 Conference in Oslo and Osteoarthritis Research Society International.

## Funding

This paper presents independent research funded by the National Institute for Health Research (NIHR) Programme Grant (RP-PG-0407-10386) and the Arthritis Research UK Centre in Primary Care grant (Grant Number 18139). KSD, ELH and CJ are part-funded by the NIHR Collaborations for Leadership in Applied Health Research and Care West Midlands. JJE was supported by an In-Practice Fellowship from the NIHR. KSD is part funded by a Knowledge Mobilisation Research Fellowship (KMRF-2014-03-002) from the NIHR. EMH is an NIHR Senior Investigator. KSD also received a grant from EIT-Health for implementation. The views expressed in this paper are those of the authors and not necessarily those of the NHS, the NIHR or the Department of Health.

## Trial registration

Trial registration number ISRCTN06984617. Trial registration status on the Register is ‘retrospective’ but recruitment of the first patient into the cluster RCT is clearly recorded on the Register as occurring on 11/05/2012, a date after the registration date of July 2011 (see Registry entry update 11/07/2016).
