# Supplementary material for: Effect of a model consultation informed by guidelines on recorded quality of care of osteoarthritis (MOSAICS): a cluster randomised controlled trial in primary care
Source: Osteoarthritis Cartilage. 2017 Oct;25(10):1588–97. doi: 10.1016/j.joca.2017.05.017 (PMC5613776; doi:10.1016/j.joca.2017.05.017)
Supplement: Supplementary file 1 [file mmc1.docx]

Appendix table 1 – Comparison between intervention and control arms of recorded quality indicator achievement from e-template – consulters with at least one e-template entry only

|  |  | Baseline period | | Trial period | |  |
| --- | --- | --- | --- | --- | --- | --- |
| Domain |  | Intervention  *n*^a^ (%) | Control  *n*^a^ (%) | Intervention  *n*^a^ (%) | Control  *n*^a^ (%) | OR^b^  (95% CI) |
|  | No. of consulters | 728 | 419 | 635 | 330 |  |
| Assessment | Pain assessment | 707 (97) | 390 (93) | 617 (97) | 318 (96) | - |
|  | Function assessment | 691 (95) | 384 (92) | 611 (96) | 309 (94) | - |
| Core management | Information given | 578 (79) | 274 (65) | 554 (87) | 268 (81) | 1.36 (0.59, 3.13) |
|  | Exercise advice | 582 (80) | 285 (68) | 526 (83) | 246 (75) | 1.88 (0.93, 3.79) |
|  | Weight loss advice^c^ | 325 (68) | 159 (57) | 341 (76) | 136 (66) | 1.29 (0.55, 3.01) |
| Non-pharmacological management | Physiotherapy referral considered | 426 (59) | 192 (46) | 348 (55) | 173 (52) | 0.81 (0.40, 1.63) |
| Pharmacological management | Paracetamol considered | 625 (86) | 349 (83) | 554 (87) | 284 (86) | 2.01 (0.91, 4.41) |
|  | Topical NSAID considered | 540 (74) | 295 (70) | 501 (79) | 275 (83) | 1.14 (0.51, 2.54) |

^a^ number of patients with record of achievement of indicator; ^b^adjusted for age, gender, coded OA or joint pain, practice level of achievement in baseline period and accounting for clustering by clinician, reference is control group; ^c^  In those recorded as overweight: baseline period intervention *n* = 478, control *n* = 280; trial period intervention *n* = 447, control *n* = 205. NSAID = non-steroidal anti-inflammatory drug

Appendix table 2 – Comparison between intervention and control arms of recorded quality indicator achievement using routinely recorded information – new consulters only

|  |  | Baseline period^a^ | | Trial period | |  |
| --- | --- | --- | --- | --- | --- | --- |
| Domain |  | Intervention  *n*^b^ (%) | Control  *n*^b^ (%) | Intervention  *n*^b^ (%) | Control  *n*^b^ (%) | OR^c^ (95% CI) |
|  | No. of consulters | 981 | 749 | 744 | 579 |  |
| Assessment | Weight record | 278 (27) | 154 (18) | 192 (26) | 107 (18) | 1.05 (0.60, 1.83) |
|  | X-ray requested | 250 (25) | 22 (3) | 118 (16) | 42 (7) | 0.34 (0.09, 1.27) |
| Non-pharmacological management | Physiotherapy referral | 90 (9) | 35 (4) | 72 (10) | 13 (2) | 5.56 (1.76, 17.52) |
| Pharmacological management | Paracetamol prescribed | 164 (16) | 155 (19) | 149 (20) | 72 (12) | 1.80 (1.18, 2.74) |
|  | Topical NSAID prescribed | 267 (26) | 194 (23) | 210 (28) | 128 (22) | 1.11 (0.71, 1.72) |
|  | PPI prescribed^d^ | 63 (35) | 27 (23) | 45 (37) | 26 (32) | 1.22 (0.47, 3.16) |

^a^ baseline includes all consulters, ^b^ number of patients with record of achievement of indicator. ^c^adjusted for age, gender, coded OA or joint pain, practice level of achievement in baseline period and accounting for clustering by clinician, reference is control group; ^d^ on date of NSAID prescription in those prescribed oral NSAIDs during time period, baseline period intervention *n* = 181, control *n* = 119; trial period intervention *n* = 121, control *n* = 81. NSAID = non-steroidal anti-inflammatory drug; PPI = proton pump inhibitor

Appendix table 3 – Comparison between intervention and control arms of recorded quality indicator achievement using routinely recorded information in those coded with osteoarthritis diagnosis

|  |  | Baseline period | | Trial period | |  |
| --- | --- | --- | --- | --- | --- | --- |
| Domain |  | Intervention  *n*^a^ (%) | Control  *n*^a^ (%) | Intervention  *n*^a^ (%) | Control  *n*^a^ (%) | OR^b^ (95% CI) |
|  | No. of consulters | 419 | 195 | 501 | 242 |  |
| Assessment | Weight record | 138 (33) | 33 (17) | 187 (37) | 34 (14) | 3.07 (1.37, 6.90) |
|  | X-ray requested | 83 (20) | 2 (1) | 59 (12) | 12 (5) | 2.35 (0.60, 9.24) |
| Non-pharmacological management | Physiotherapy referral | 38 (9) | 5 (3) | 37 (7) | 2 (0.8) | 8.84 (1.07, 72.68) |
| Pharmacological management | Paracetamol prescribed | 92 (22) | 49 (25) | 143 (29) | 39 (16) | 2.06 (1.34, 3.17) |
|  | Topical NSAID prescribed | 128 (31) | 44 (23) | 180 (36) | 68 (28) | 1.26 (0.77, 2.07) |
|  | PPI^c^ prescribed | 30 (41) | 13 (36) | 32 (44) | 18 (45) | 1.06 (0.47, 2.41) |

^a^ number of patients with record of achievement of indicator, ^b^ adjusted for age, gender, practice level of achievement in baseline period and accounting for clustering by clinician, reference is control group; ^c^ on date of NSAID prescription in those prescribed oral NSAIDs during time period, baseline period intervention *n* = 74, control *n* = 36; trial period intervention *n* = 72, control *n* = 40. NSAID = non-steroidal anti-inflammatory drug; PPI = proton pump inhibitor

**Appendix 1 The model OA consultation**

A model OA consultation provided a new service for patients in general practice to enhance the management of OA based on the NICE OA guidelines (NICE 2008, 2014). The model consultation was a linked GP and practice nurse integrated consultation supported by the use of an OA Guidebook. The aim of the model OA consultation was to operationalise in general practice three aspects of care for OA:

1. The three core treatments of the NICE OA Guideline (2008): verbal and written information, advice to exercise and increase physical activity, interventions to achieve weight loss.
2. The NICE OA Guideline recommendations for first-line analgesia: paracetamol and topical NSAIDs.
3. Support for self-management of OA based upon the Whole Systems Informing Self-Management Engagement (WISE) model (Kennedy et al, 2007), which centres on the provision of knowledge for patients and a style of intervention built on professional responsiveness to patients’ needs.

**Model OA consultation with the GP**

Patients aged ≥45 years with peripheral joint pain (knee, hip, hand, and foot) had an initial consultation with the GP. An OA e-template was triggered as part of the consultation and GPs were asked to assess and make a clinical diagnosis of the problem without the routine use of x-ray. For patients diagnosed with OA, GPs were then asked to offer an explanation of OA (in suitable language and tailored to the patient's level of understanding and individual circumstances) and offer first line analgesia as appropriate (paracetamol; topical NSAIDS). An OA guidebook (weblink: http://www.keele.ac.uk/media/keeleuniversity/ri/primarycare/pdfs/OA_Guidebook.pdf) written by patients and health care professionals for patients was given to the patient. It offers support for self-management, promotes the NICE core treatments and provides accounts of how people live with OA. The GP was then asked to explain the next steps: for the patient to read the OA guidebook and to arrange a follow-up appointment with the practice nurse.

**Model OA consultation with the practice nurse (nurse-led OA clinic)**

The timing of the first appointment with the practice nurse was planned for a minimum of two weeks after the initial GP consultation. This gave patients time to read the guidebook and try self-management strategies they felt were suitable. In the first consultation the practice nurse was asked to refer to the guidebook as a resource to answer questions and clarify issues, ascertain the advice from the GP consultation, negotiate and agree appropriate goals, discuss the need for pain relief and opportunities for healthy eating, physical activity and exercise as appropriate.

The timing of up to three follow-up visits with the nurse was agreed between the patient and the practice nurse, but was scheduled to be delivered within three months following the GP consultation. The follow-up practice nurse consultations were tailored to the patient’s individual needs and could focus on, for example, reviewing the self-management plan, demonstrating exercises (Arthritis Research UK Exercises for Arthritis leaflet), giving advice as to how this could be maintained longer-term or making any necessary referrals to the broader multidisciplinary team. The practice nurse consultations were supported by a specifically tailored Case Report Form (available on request) and a nurse toolkit that included advice leaflets to give to patients (content of the toolkit available on request).

The figure below provides an overview of the model OA consultation.

Appendix References

1. National Institute for Health and Care Excellence. Osteoarthritis: the care and management of osteoarthritis in adults. National Institute for Health and Care Excellence, 2008.
2. National Institute for Health and Care Excellence. Osteoarthritis: care and management in adults. National Institute for Health and Care Excellence, 2014.
3. Kennedy A, Rogers A, Bower P. Support for self care for patients with chronic disease. *BMJ* 2007;**335**:968-70.
