# Supplementary material for: Effect of a model consultation informed by guidelines on recorded quality of care of osteoarthritis (MOSAICS): a cluster randomised controlled trial in primary care
Source: Osteoarthritis Cartilage. 2017 Oct;25(10):1588–97. doi: 10.1016/j.joca.2017.05.017 (PMC5613776; doi:10.1016/j.joca.2017.05.017)
Supplement: Supplementary file 2 [file mmc2.pdf]

Dziedzic et al. Implementation Science 2014, 9:95
http://www.implementationscience.com/content/9/1/95

IMPLEMENTATION SCIENCE

STUDY PROTOCOL

Open Access

Implementing the NICE osteoarthritis guidelines: a mixed methods study and cluster randomised trial of a model osteoarthritis consultation in primary care - the Management of OsteoArthritis In Consultations (MOSAICS) study protocol

Krysia S Dziedzic[1], Emma L Healey[1*], Mark Porcheret[1], Bie Nio Ong[1], Chris J Main[1], Kelvin P Jordan[1], Martyn Lewis[1], John J Edwards[1], Clare Jinks[1], Andrew Morden[1], Gretl A McHugh[2], Sarah Ryan[3], Andrew Finney[1], Sue Jowett[1,4], Raymond Oppong[1,4], Ebenezer Afolabi[1], Angela Pushpa-Rajah[5], June Handy[1], Kris Clarkson[1], Elizabeth Mason[1], Tracy Whitehurst[1], Rhian W Hughes[1], Peter R Croft[1] and Elaine M Hay[1]

Abstract

Background: There is as yet no evidence on the feasibility of implementing recommendations from the National Institute of Health and Care Excellence (NICE) osteoarthritis (OA) guidelines in primary care, or of the effect these recommendations have on the condition. The primary aim of this study is to determine the clinical and cost effectiveness of a model OA consultation (MOAC), implementing the core recommendations from the NICE OA guidelines in primary care. Secondary aims are to investigate the impact, feasibility and acceptability of the MOAC intervention; to develop and evaluate a training package for management of OA by general practitioners (GPs) and practice nurses; test the feasibility of deriving 'quality markers' of OA management using a new consultation template and medical record review; and describe the uptake of core NICE OA recommendations in participants aged 45 years and over with joint pain.

Design: A mixed methods study with a nested cluster randomised controlled trial.

Method: This study was developed according to a defined theoretical framework (the Whole System Informing Self-management Engagement). An overarching model (the Normalisation Process Theory) will be employed to undertake a comprehensive 'whole-system' evaluation of the processes and outcomes of implementing the MOAC intervention. The primary outcome is general physical health (Short Form-12 Physical component score [PCS]) (Ware 1996). The impact, acceptability and feasibility of the MOAC intervention at practice level will be assessed by comparing intervention and control practices using a Quality Indicators template and medical record review. Impact and acceptability of the intervention for patients will be assessed via self-completed outcome measures and semi-structured interviews. The impact, acceptability and feasibility of the MOAC intervention and training for GPs and practice nurses will be evaluated using a variety of methods including questionnaires, semi-structured interviews, and observations.

(Continued on next page)

* Correspondence: e.healey@keele.ac.uk
[1]Arthritis Research UK Primary Care Centre, Keele University, Keele, Staffordshire ST5 5BG, UK
Full list of author information is available at the end of the article

BioMed Central

© 2014 Dziedzic et al.; licensee BioMed Central Ltd. This is an Open Access article distributed under the terms of the Creative Commons Attribution License (http://creativecommons.org/licenses/by/4.0), which permits unrestricted use, distribution, and reproduction in any medium, provided the original work is properly credited. The Creative Commons Public Domain Dedication waiver (http://creativecommons.org/publicdomain/zero/1.0/) applies to the data made available in this article, unless otherwise stated

(Continued from previous page)

Discussion: The main output from the study will be to determine whether the MOAC intervention is clinically and cost effective. Additional outputs will be the development of the MOAC for patients consulting with joint pain in primary care, training and educational materials, and resources for patients and professionals regarding supported self-management and uptake of NICE guidance.

Trial registration: ISRCTN number: ISRCTN06984617.

Keywords: Osteoarthritis, General practice, Implementation, Primary care, NICE guidelines, Self-management

Background

There is a perception that osteoarthritis (OA) is a 'natural' part of ageing and there are limited interventions available [1,2]. Whilst there are many published guidelines on the treatment of OA [3-10] there is a gap between the care that is recommended and the care that patients receive [11,12]. NICE have recommended that all patients with OA should be offered three core treatments when they first present in primary care (see Figure 1): education and access to information; advice on local muscle strengthening exercise and general aerobic fitness; and, if appropriate, advice on losing weight [13].

For patients and healthcare professionals (HCPs) in primary care, the clear message that emerges from the guidelines is that there is a range of simple interventions for which there is evidence of clinical effectiveness. By contrast, there is clear evidence that the core aspects of assessment and management of OA as currently delivered in primary care do not meet the recommendations of these guidelines [11]. Two recent Delphi consensus exercises have been conducted to address the limited research evidence on the content of a model OA consultation during which the core package of care could be delivered [14,15]. However, there is as yet no evidence on the feasibility of using this model consultation as a way of implementing NICE core OA treatments in primary care and the effect of this support for self-management on the course and impact of the condition.

The importance of self-management for long term illnesses and of professional support for self-management are emphasised in NHS policy [16]. Studies have shown that among patients consulting for knee OA, core treatments were mostly self- rather than doctor-initiated [1,11]. Previous research suggests that patients need more information about OA to enable them to manage their condition more effectively [17]. Lay ideas of self-management include minimising the impact of conditions [18,19], maintaining a sense of 'normality' [18,20], and maintaining social roles and obligations [21,22].

The way in which complex interventions, such as supported self-management approaches, are developed and then become embedded in routine clinical practice needs to be supported by theoretical models and evaluated appropriately [23]. A specific model of support for self-management called the 'Whole system Informing Self-management Engagement' (WISE) model [24] is predicated upon the argument that for self-management support

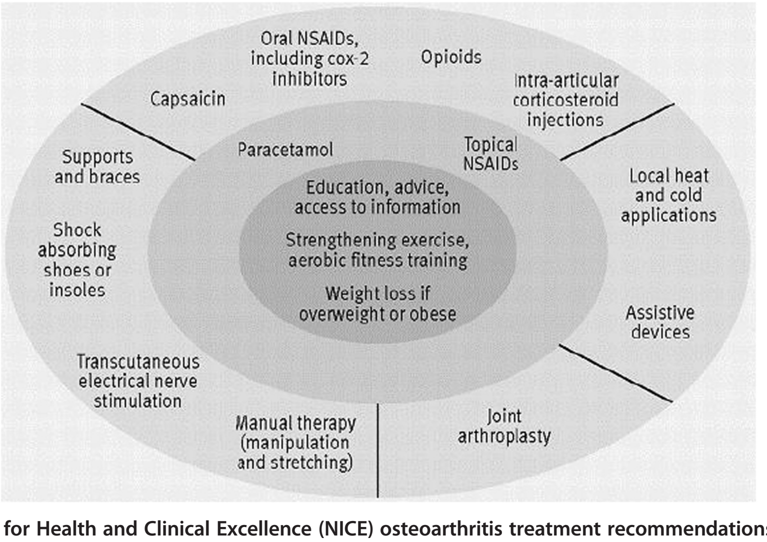

Figure 1 National Institute for Health and Clinical Excellence (NICE) osteoarthritis treatment recommendations (Conaghan et al. [10]). Reproduced from, Care and management of osteoarthritis in adults: summary of NICE guidance, Conaghan P, Dickson J, Grant R, 336, 502-503, 2008, with permission from BMJ Publishing Group Ltd.

to be effective, it requires the understanding and incorporation of the patient agenda into the consultation, including what they are already doing. A whole systems approach is needed, which engages with practitioners and service organisations as well as the patient [24]. The WISE model has been used in a number of long-term illnesses [25-27], and envisages informed patients receiving support and guidance from trained practitioners who are working within a healthcare system that is geared up to be responsive to patients' needs.

Michie and colleagues have produced a synthesis of psychological theories to enable design and implementation of behaviour change interventions [28-30], and Grol and colleagues have developed a useful framework for translating evidence into practice [31]. The Calgary-Cambridge framework has specifically been developed to enhance consultation skills [32]. The Normalisation Process Theory (NPT) [33,34] is a sociological theory concerned with understanding and evaluating how complex interventions become embedded in routine clinical practice. These approaches will be integrated to develop the model OA consultation (MOAC) intervention and the HCP training in this study.

This study is focused on determining the current management of OA in consultations and whether supported self-management, delivered in a model OA consultation, could offer a clinically practicable approach to implementing the core NICE recommendations. We will undertake a cluster randomised controlled trial (RCT) to evaluate the whole systems approach and to determine the clinical and cost effectiveness of the MOAC intervention. The protocol has been reported using the SPIRIT recommendations [35,36].

Aims

The primary aim of the study is to:

1. Determine the clinical and cost effectiveness of the MOAC intervention in patients with OA.

Secondary aims are to:

1. Describe the uptake of core NICE OA recommendations in participants aged 45 years and over with joint pain;
2. Test the feasibility of deriving 'quality markers' of OA management using a new consultation template and medical record review;
3. Develop and evaluate a training package for management of OA by general practitioners (GPs) and practice nurses;
4. Investigate the impact, feasibility and acceptability of the MOAC intervention.

Design

The MOSAICS study is a mixed methods study with a nested cluster RCT based in eight general practices, comprising of four components (see Figure 2).

Participants

HCPs and their respective practice populations from eight general practices will be invited to participate and randomly allocated to two clusters: 'intervention' and 'control' practices. The practice populations of the practices recruited, aged 45 years and over, will form the sampling frame for the overall study. The eligibility criteria for the study are described in Table 1. Resources to support primary care engagement are described in Additional file 1.

Ethics approval

The study has been approved by the North West 1 Research Ethics Committee, Cheshire (REC reference: 10/H1017/76).

Consent

Informed consent will be obtained from all HCPs and patients participating in the study (see Additional file 2).

Components of the MOSAICS study

1. The cluster randomised controlled trial
2. Population survey
3. Consultation template and medical record review
4. Evaluation of the MOAC intervention and the training

Study component 1: The cluster RCT

This will be a two-arm prospective pragmatic cluster RCT based in eight general practices in the North West midlands and Cheshire, UK. This trial will test the hypothesis that the MOAC intervention is superior to control and will be a parallel group longitudinal design with repeated measures. Practices will be randomly allocated to two clusters: 'intervention' and 'control' practices. Stratified block randomisation will be performed with the practices stratified into four largest and four smallest practices by list size. In each stratum, the practices will be randomly allocated by a computerised random number generator using blocking with two practices per study group.

Recruitment to the trial will occur over a six-month period. All practices will use the OA template and will be given standard information on OA for patients and healthcare professionals published by Arthritis Research UK. In four practices, patients will receive the MOAC intervention, while the other four practices will continue to provide usual care. GPs in the intervention arm will be offered training for the initial consultation (MOAC 1), and practice nurses

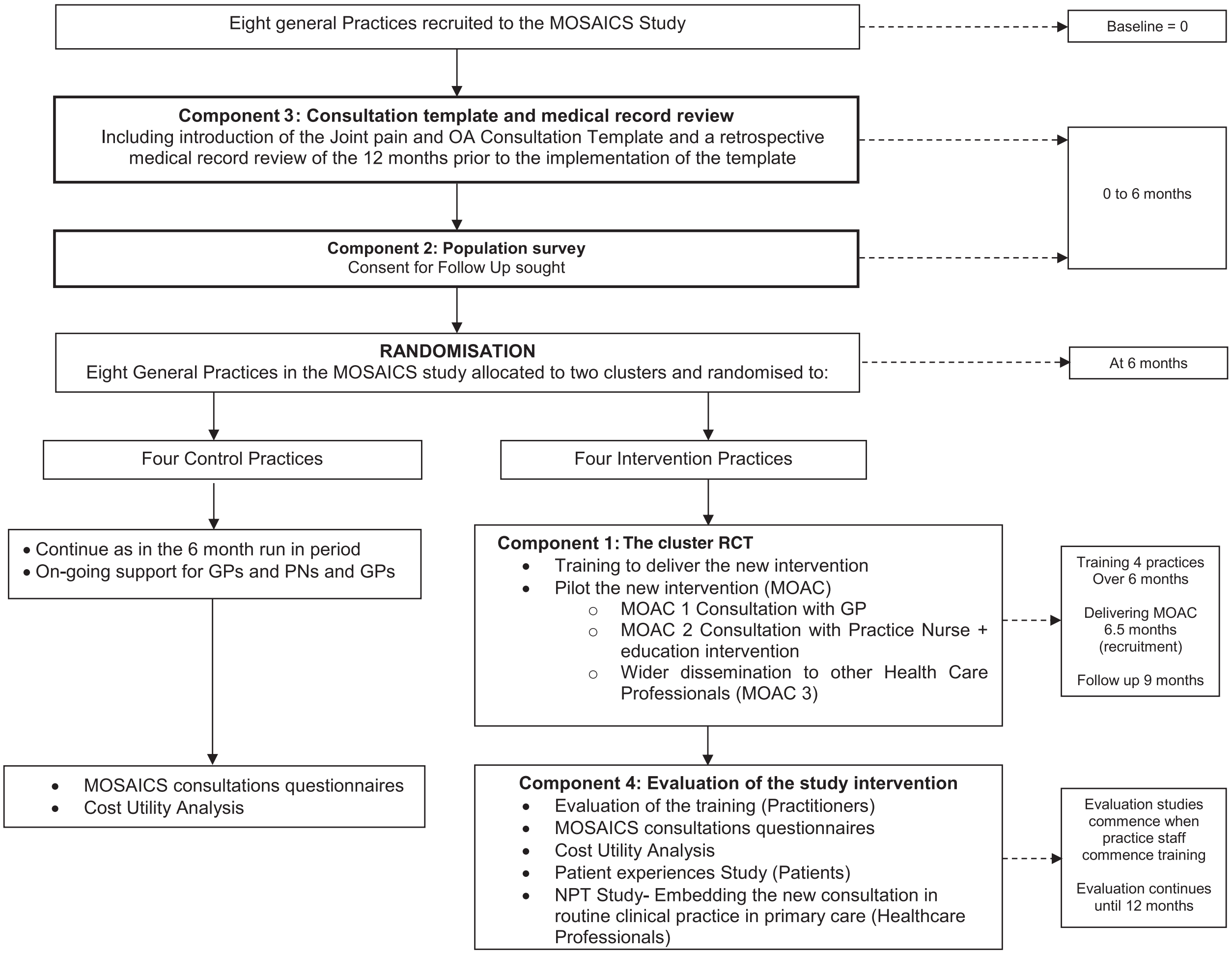

Figure 2 MOSAICS study overview.

will be offered comprehensive training for follow-up consultations (MOAC 2), to deliver supported self-management of OA. The control practices will have the opportunity to receive the training at the end of data collection.

The intervention

The MOAC intervention

In order to identify the content of the MOAC intervention, two consensus exercises with GPs, lay participants, practice nurses, community pharmacists, and allied health professionals (occupational therapists, physiotherapists, podiatrists) have been conducted [14,15]. Using the findings of these exercises and theoretical models to guide self-management (WISE) and support patient behaviour change, a three-stage consultation has been proposed. The MOAC intervention comprises of MOAC 1 (initial consultation with GP), MOAC 2 (follow-up visits with practice nurse) [37], and MOAC 3 (dissemination to other HCPs). Consultations will be recorded in the general practices through the use of the joint pain and OA consultation template (Additional file 3 provides the full details of the intervention).

The OA Guidebook

The Arthritis Research UK Primary Care Centre at Keele University has developed a guidebook for patients and professionals to use as an aid to support self-management for OA, and this will be used in MOAC [38]. The OA Guidebook contains both evidence-based biomedical information and lay experiential knowledge about the nature of OA, how it is diagnosed and treated, and the ways in which people who have OA continue to keep going in their everyday lives. Together these will give patients insight into the rationale for the advice and treatments offered in MOAC 1, 2 and 3 and what, practically, people who have OA have found helpful in learning to live with the condition. The patient guidebook will be a useful way to reinforce verbal information given by HCPs, to act as a resource to consult if queries or uncertainties arise in the future, to help

Table 1 MOSAICS study eligibility criteria

Eligibility criteria	
**General practices and health-care professionals**	• Member of the Central England PCRN or a Keele Research Network Practice
	• At least two GPs willing to undertake the study as per protocol, *i.e.*, act as a control or intervention practice
	• Willing, and able, to allow one (or for preference two – to allow for cross cover) of their practice nurses to be trained to deliver the MOAC 2 clinics
	• Able to physically accommodate the nurse clinics in the practice
	• Uses the EMIS computerised consultation system
	• Nurses and GPs consenting to follow up by the MOSAICS study team
	• GPs willing to be trained to carry out the MOAC 1 consultations
	• Nurses willing to be trained to carry out the MOAC 2 clinics
	• Nurses who consent to being observed and audio recorded in MOAC 2 clinics
***Patients**	Inclusion Criteria:
	• Males and Females
	• 45 years and over
	• Registered with a MOSAICS study practice
	• Consenting to further contact from the study team and medical record review (consent sought as part of the Patient Population Survey)
	Exclusion Criteria:
	• Excluded via GP screen of practice list
	• Unable to give fully informed consent, *e.g.*, learning difficulties or dementia
	• Resident in a care or nursing home
	• History of serious disease, *e.g.*, malignancy, terminal illness
	• Unable to consult in the general practice
	• Flagged as excluded from research in that practice

EMIS = Egton Medical Information System; GP = General Practitioners; MOAC = Model Osteoarthritis Consultation; MOSAICS = Management of Osteoarthritis In consultations; OA = Osteoarthritis; PCRN = Primary Care Research Network.
*All patients registered with MOSAICS practices randomised to the intervention arm of the study had access to the MOAC 1 and MOAC 2 clinics, including those who did not consent to be followed up, as these ran as part of normal clinical care within each intervention practice.

structure MOAC 2 consultations, and to prompt questions to ask HCPs.

The training

Training and educational packages will be developed for GPs and practice nurses by drawing on the work of Michie et al. [29,30]. GPs will receive training on how to deliver the initial consultation (MOAC 1) for a new or established patient consulting with OA and the procedure for referring to a practice nurse for a follow-up OA consultation (MOAC 2). Practice nurses will receive training in how to support and enable patients to self-manage OA, using a patient-centred approach, an OA guidebook, goal setting, pain management and the core NICE recommendations (information and advice, strengthening exercise and aerobic fitness training, and weight management). Nurses will also receive training in how to complete the MOAC 2 case report form (CRF), which will be completed for each patient attending the MOAC 2 consultations. Full details of these training packages will be published separately.

For MOAC 3, members of the wider multidisciplinary team linked to the intervention practices (*e.g.*, physiotherapists, occupational therapists, podiatrists, pharmacists) will be invited to workshops to increase awareness of the study and its aims.

Sample size

Published research in musculoskeletal disorders has estimated a minimal clinically important difference (MCID) from 2 to 4 points for the SF-36 version 2 Physical Component Summary (PCS) subscale [39], which has a normalised population standard deviation (SD) of 10. Hence, a difference between groups of 0.3 of an effect size is considered a minimum clinically important difference (MCID) threshold for the purposes of demonstrating superiority in the MOSAICS RCT.

In total, 500 patients will need to be recruited in expectation that 400 will provide data at six months. A total of 400 participants (200 to each arm) will ensure 90% power to detect at least the effect size of 0.3 at the primary time point of six months follow-up given a 5% two-tailed significance level. Randomisation is by practice, so this sample size calculation was inflated to correct for an intracluster correlation coefficient (adjusted ICC of 0.005), varying practice size recruitment was taken into account (including coefficient of variation of 0.5 as per POST trial [ISRCTN40721988]) and inclusive of (×0.67) and × 1.25 respective adjustments for repeated-measures design and 20% dropout allowance.

Patient level evaluation

Patient consultation questionnaire

Eligible participants who consented to medical record review and further contact in the initial baseline patient population survey (see component 2), and who subsequently consult with joint pain in one of the four intervention practices will be identified via fortnightly electronic searches of consultation data and receive a consultation questionnaire asking about their consultation experience with the MOAC intervention. This will also be

administered to eligible participants in the four control practices, to enable comparison of patient reported clinical outcomes.

The primary clinical outcome measure for the cluster RCT is the SF12 PCS [40]. The primary follow-up time point is six months. Key secondary outcomes include the Arthritis Self-Efficacy pain subscale [41] and the OMERACT/OARSI responder criteria [42]. Other outcome measures collected in the trial can be found in (Table 2). Participants will be followed up at 3, 6 and 12 months after consultation to determine short, medium and long term behaviours and patterns of the uptake of core OA treatments. Process outcome measures will also be collected within the study (*e.g.*, achieving Quality Indicators of care).

The trial statistician will be kept blind to the practice allocation until after the analysis of the primary and secondary outcomes (blinding will be broken for the per protocol analysis) [43].

Objectives: Study component 1

1a. To determine the clinical and cost effectiveness of the MOAC intervention in four general practices compared with four control practices using patient reported outcome measures.
1b. To train healthcare professionals (GPs and practice nurses) to deliver the MOAC intervention.
1c. To implement the MOAC intervention to deliver supported self-management for adults with OA within routine primary care.

Trial status

Eight general practices have been recruited, and the intervention has been developed and delivered in the four intervention practices. We expect follow-up data collection to be complete in 2014.

Study component 2: Population survey

A cross sectional population survey will be mailed to an estimated sample of 30,000 adults aged 45 years and over registered in the eight participating general practices participating in the study. After exclusions and based on previous similar studies from the Research Centre, we anticipate a sample of 9,600 with self-reported joint pain.

The population survey will use a 2-stage mailing process based on established procedures conducted in the Arthritis Research UK Primary Care Centre. Eligible participants will be sent a letter of invitation to take part in the survey, information about the study, and the population survey. This survey will collect demographic and work-related data and ask questions regarding general and psychological health, physical activity, joint pain in the last 12 months, consultation behaviour and the management of their joint pain (knee, hip, foot, hand) (the full list of outcome measures can be found in Table 3). Consent will be sought for further involvement in the MOSAICS study and for allowing access to their medical records. Individuals excluded by the GP or contacting the research team and not wishing to take part in the study will be tagged in the practices as exclusions and will not be contacted again for this study. After two weeks, non-responders will be sent a reminder survey and letter.

Those that respond to the population survey and agree further contact will be approached for data collection regarding the cluster RCT if they consult with joint pain during the recruitment period. The key reason for doing this is to minimise selection bias in the trial by separating the process of consent from the intervention.

Objectives: Study component 2

2a. To describe pain severity, general health, psychological status, and uptake of core treatments for OA recommended by NICE in participants aged 45 years and over with joint pain (hip, knee, hand, foot).
2b. To identify a population within the practices that agrees to further contact and medical record review and can therefore be approached for data collection for the cluster RCT.

Study component 3: Consultation template and medical record review

This component will collect anonymised practice-level data to describe the management of OA in primary care via retrospective and prospective medical record review in the eight practices recruited to the study. The OA consultation template will record specific Quality Indicators for the core management of OA which are not routinely recorded in practice. These indicators were established through a systematic review of Quality Indicators of OA [48]. These include pain and functional impairment, provision of information, advice about exercise and weight loss, and advice about pharmacological management such as use of paracetamol and topical non-steroidal anti-inflammatory drugs (NSAIDs).

A download of routine consultation, prescription and other management data related to joint pain and OA will be obtained for the preceding 12 months before the introduction of the template. Data regarding certain co-morbidities, identified as having implications for prescribing behaviours in the management of joint pain and OA, will also be obtained for three years preceding the introduction of the template.

The OA consultation template will then be introduced in all of the eight practices at baseline (six months prior to randomisation of the practices for the cluster RCT described in component 1). Training regarding use of

Table 2 Consultation survey measures

Data Collection	Measurement Scale	Time points			
		Baseline	3 months	6 Months	12 Months
Demographic Information					
Age	Years	✓	✓	✓	✓
Gender	Female/Male	✓	✓	✓	✓
Weight	Stones and lbs or Kilograms	✓	✓	✓	✓
Work related Questions					
Current/most recent job title	Free Text	✓	✓	✓	✓
Currently in a paid Job	Yes/No/Retired	✓	✓	✓	✓
Typical working week	Working full time (30 hours or more per week)/ Working part time (29 hours or less per week)/	✓	✓	✓	✓
Time off during last 6 months because of joint pain including time off to visit any health care professional	Yes/No	✓	✓	✓	✓
How many days, weeks or months were you absent from work due to joint pains in the last 6 months	Number of days/weeks/months	✓	✓	✓	✓
Consultations					
GPAQ communication sub scale [43]	8 – 56	✓			
GPAQ for Nurses communication sub scale (modified GPAQ [43])	8 – 56		✓		
Consulting Practice Nurse (single question)	Yes/No		✓		
Joint Pain					
Specific Joint Pain and Problems in knee/ hip/hand/foot over 3 months	Yes/No	✓	✓	✓	✓
Pain intensity in the knee/hip/hand/foot over 3 months	0-10 numerical rating scale	✓	✓	✓	✓
WOMAC Physical function subscale [44]	0 - 32	✓	✓	✓	✓
AIMS 2 hand and finger function subscale [45]	0-20	✓	✓	✓	✓
Physical Activity					
IPAQ [46]	Categorical score: low, moderate, high<br>Continuous score: MET-min per week	✓	✓	✓	✓
Walking Questions	Where do you regularly walk for reasons including health and well-being?/ Who do you regularly walk with?	✓	✓	✓	✓
PASE [47]	0 – 361	✓	✓	✓	✓
Global Assessment of change [48]	Completely recovered, much better, better, no change, worse, much worse		✓	✓	✓
General health and Well being					
PHQ8 [49]	0 – 24	✓	✓	✓	✓
GAD7 [50]	0 – 21	✓	✓	✓	✓
SF12 version 2 [40]	0 – 100	✓	✓	✓	✓
SF6D [51]	0.29-1				
EQ-5D [52]	-0.59-1	✓	✓	✓	✓
ICECAP-A version 2 [53]	4 - 20	✓	✓	✓	✓
Managing your joint problems					
Arthritis Self Efficacy pain sub scale [41]	0 - 10	✓	✓	✓	✓

Table 2 Consultation survey measures *(Continued)*

Patient generated OA Quality Indicators (Adapted from Østerås et al [54])	15 questions (Yes/No/Don't remember)	✓	✓	✓	✓
Medication/Treatment Use (adapted for joint problems from Jinks et al [55])	Simple count of strategies used	✓	✓	✓	✓
Patient Enablement (modified from Howie et al [56])	0 - 10		✓	✓	✓
Healthcare Utilisation	Self-help remedies, contact with NHS and private healthcare, over the counter medicines, prescribed medication			✓	✓

Key:
AIMS 2 = Arthritis Impact Measurement Scale; GAD 7= Generalized Anxiety Disorder 7; GP = General Practitioner; GPAQ = General Practice Assessment Questionnaire;
ICECAP-A = self-report measure of capability wellbeing for adults; IPAQ: International Physical Activity Questionnaire; MET = Metabolic Equivalent; PASE = Physical Activity Scale for the Elderly; PHQ8 = Patient Health Questionnaire 8; EQ5D = Quality of Life; SF12 = Short Form 12; SF6D = Short Form 6D; WOMAC = Western Ontario and McMaster Universities Arthritis Index.

the template as part of routine consultations for patients presenting with a working diagnosis of OA (knee, hip, hand or foot pain) will be provided for the GPs and practice nurses. When a patient aged 45 or over with joint pain consults in any of the eight practices and the GP or practice nurse enters a Read code for OA or one of a selection of joint pain Read codes (see Additional file 4), the OA consultation template will open to allow data entry. The GP or practice nurse undertaking the consultation may complete the template, or bypass the template, as appropriate (for various reasons, such as the joint pain not being related to OA). Data on routine recording of management and Quality Indicators will be collected in the template prospectively at 6 months after baseline and 21 months by downloading anonymised practice data.

Objectives: Study component 3

3a. To describe the current recording patterns and management of OA in primary care based on a 12-month retrospective download of medical records.
3b. To describe change in the recording patterns and management of OA in primary care following (i) activation of the template, and (ii) delivery of the MOAC intervention, based on prospective downloads of medical records.

Study component 4: Evaluation of the MOAC intervention and the training

This component will evaluate (i) the implementation of the MOAC intervention at the level of the service, HCPs and patients, and (ii) the training. Both qualitative and quantitative methods will be used.

Evaluation of the intervention

Practice level evaluation

Observations of a number of relevant meetings will be undertaken. The number of observations will be decided, in negotiation with each intervention practice. The intention is to observe all relevant clinical, management and research meetings to reveal how the intervention is operationalised within practice. In order to document events, observations will be structured and will use audio recordings and an observation framework to capture data for analysis. Secondary data collection will also be undertaken to determine adherence to the intervention. For example, notes of meetings in the practices, MOAC 2 CRFs, patient records, and patient feedback forms will be used to gain an understanding of how the intervention was taken up and delivered.

HCP level evaluation

In order to capture the complexity of how the intervention is received, delivered and managed by members of the primary healthcare team, a range of methods will be used. These methods include semi-structured face-to-face interviews with HCPs, structured observations of consultations, video-recorded simulated consultations, and secondary data collection (for example, minutes of meetings).

GP and practice nurse interviews and focus group

As part of the last training session, all GPs and practice nurses will be invited to participate in a focus group, to ascertain whether, and if so how, their views of OA treatment have changed since the training, and to explore what this means for their clinical practice. GPs and nurses from the intervention practices will also be invited to participate in individual telephone or face-to-face interviews after completion of the intervention. All interviews will be digitally-recorded and fully transcribed and conducted six to nine months following randomisation.

Practice nurse observations

Observations of MOAC 2 consultations will be undertaken to gain detailed insight into the consultation itself, the interaction between the nurse and patient, and the actual delivery of MOAC 2 in routine practice. Audio recordings and detailed field notes will capture data for

Table 3 Population survey outcome measures

Data Collection	Measurement Scale
Demographic	
Age	Years
Gender	Female/Male
Marital status	Married/separated/divorced/widowed/cohabiting/single
Living alone	Yes/No
Spouse/Partner cohabiting	Yes/No
Cost of living	Find it a strain to get by from week to week/Have to be careful with Money/Able to manage without much difficulty/Quite comfortably off
Height	Feet and inches or centimetres
Weight	Stones and lbs or kilograms
Work related questions to determine occupational social class	
Employment status	Employed / Not working due to ill health /Retired/Unemployed or seeking work/ Housewife/Other
Spouse/partner's job title	Free Text
Work related questions including:	Current employment status; Job title (or previous job title if retired/unemployed); Spouse's job title (or previous job title if no longer working or deceased)
General health	
SF12 version 2 (physical and mental summary score) [40]	0 - 100
GAD7 [50]	0 - 21
EQ-5D 3 level version [52]	-0.59 - 1
Physical Activity	
STAR [58]	Three categories: 1) Physically inactive, 2) Meets the current recommendations of physical activity, 3) Insufficiently active
Joint Pain	
Specific joint pain and problems in knee/hip/hand/foot over past year	Yes/No
Pain intensity in the knee/hip/hand/foot over past month	0-10
Managing Joint Problems over the last 12 months	
Consultation with practice nurse (single question)	Yes/No
Consultation with GP (single question)	Yes/No
Medication/Treatment use (adapted for joint problems from Jinks et al [55])	Simple count of strategies used

GP = General Practitioner; GAD 7 = Generalized Anxiety Disorder 7; EQ5D = Quality of Life; SF12 = Short Form 12; STAR = Short Telephone Activity Rating.

analysis and allow verbal and non-verbal communications to be compared.

Video-recorded simulated patient consultations

To evaluate OA consultation behaviour, the GPs in the intervention practices will be invited to undertake video-recorded consultations with simulated patients. The simulated patient will take on the role of one with chronic joint problems, and the GP will be asked to conduct a consultation in which the problems are assessed and a management plan agreed upon. Video-recorded consultations will be undertaken (i) before the training (video 1), (ii) during training (video 2), (iii) one month after training (video 3), and (iv) six months after the training (video 4). Two video-recorded consultations (videos 1 and 2) will be used as part of the training to enable GPs to reflect on their consultation behaviours and video-recorded consultations. Videos 1, 3 and 4 will be used to evaluate change of behaviour after training. Video-recorded consultations will be assessed by four independent and blinded (to the time-point of the video-recording) raters, using a pre-defined rating tool.

Participant level evaluation

Patient interviews

These interviews will be carried out within the four intervention practices. In-depth semi-structured interviews will be undertaken to explore patients' experiences of

the MOAC intervention. The purpose is to understand whether the advice and support offered is relevant to patients, whether they have implemented any of the recommendations, and how this has affected their perception of OA and its management.

For MOAC 1, individual in-depth interviews will be carried out in order to ascertain people's personal experiences and perspectives of their OA and the consultation. For MOAC 2, dependent on patient preference, either individual or group interviews will be held - bringing together three or four participants per group. The main reason for choosing the group interview is to avoid people feeling that they are being 'checked up on' with regards to implementing the advice and support offered in MOAC 2. The discussion within a group setting allows for the focus to be on the relevance and adoption of the intervention rather than on individuals.

The sampling frame for this study will be the MOSAICS consultation questionnaire described previously. In order to explore a range of accounts and behaviours, purposive sampling is required. Further variation will be achieved by selecting patients according to type of pain, age and gender. All interviews will be digitally-recorded and fully transcribed.

Two groups of patients will be purposively sampled:

1. Patients who have attended MOAC 1 will be interviewed soon after they have consulted their GP for their joint problem. An interview guide will provide a flexible framework for questioning, asking, for example; how do you feel the consultation went? Have you done anything differently? What information was given by the GP, and was it relevant/appropriate? How did you feel about being referred to a nurse? Approximately 15 people will be interviewed about their experiences of receiving MOAC 1.
2. Patients who have attended MOAC 2 will, dependent upon patient preference, either be interviewed individually or in small groups after completing up to four consultations with the nurse and returning their three-month consultation questionnaire. An interview guide will also be used and will focus questions around the expectations of the nurse consultation and what happened during the consultation, how the guidebook was used and how they felt about it, and overall what worked and what didn't. Approximately 15 people will be interviewed about their experience of receiving MOAC 2. These 15 people may not be the same as those interviewed about MOAC 1.

Evaluation of the training

Training evaluation questionnaires

Before training, to explore drivers for participation in the study, all GPs and practice nurses in participating practices will be invited to answer two open-ended questions that will focus on (i) their reasons for participating in the study and (ii) the perceived benefits (for clinical practice, for the primary care organisation, for their own role etc.). A baseline questionnaire assessment of knowledge about and beliefs and attitudes to OA and its management in primary care, will also be conducted. We will ask the GPs and practice nurses in the intervention practices who have received training to complete two further questionnaires (one month and six months post-training) to determine any change in knowledge, beliefs and attitudes following the training.

All GPs and practices nurses from the control practices will have the opportunity to attend the training at the end of the study. They will also be asked to complete the same evaluation questionnaire as the intervention practice staff at baseline (pre-training) and one month after the training (post-training).

Health economics evaluation

The economic evaluation will provide a preliminary analysis of the cost effectiveness (cost-utility) of the template and MOAC intervention compared with template alone, over a 12-month period.

Objectives: Study component 4

4a. To determine whether the training changes health care professionals' behaviour.
4b. To explore change in recording in medical records, and the uptake of core treatments for OA recommended by NICE in all participants consulting with joint pain (hip, knee, hand, foot) and the subgroup coded as OA by the general practitioner, following the MOAC intervention.
4c. To explore the cost-effectiveness of the template and MOAC intervention compared with the template alone.
4d. To explore patient experiences of their consultation for joint pain and whether the new intervention (MOAC) is acceptable and feasible in primary care (qualitative work).
4e. To examine and evaluate the way in which a new consultation for OA can be embedded in routine clinical practice in primary care (implementation).

Analysis

Analysis of the cluster RCT

Baseline characteristics will be compared between treatment arms and presented at the level of: (i) GP-Practice clusters, and (ii) Patient characteristics.

Baseline data for GP-Practice characteristics will include data on the stratification variables for randomisation – *i.e.*, practice list size and the number of GP practitioners, median index level of deprivation for the Practice, mean

age and gender profile of the Practice populations. Baseline data for patients will include data pertaining to participants' demographic characteristics, joint problem, management of their joint problem, general health and quality of life.

Balance of baseline characteristics is particularly important to establish for cluster trials given the higher level unit of randomisation and thus the potential for bias in the selection and recruitment uptake of patients. No formal statistical testing will be carried out for differences in baseline characteristics except for the GPAQ communication sub scale [49], as this is measure of the uptake of the model OA consultation by GPs.

Descriptive statistics on mean scores for numerical outcomes and frequency counts and percentages for categorical data will be presented for outcome measures from the consultation questionnaire, stratified by study group (intervention or control). A Linear Mixed Model will be used to analyse primary outcome data (SF-12 PCS). Statistical testing of clinical and process outcomes between study groups will be performed using regression methods (adjusting for age, gender, baseline SF12-PCS and corresponding baseline value, of the outcome being measured, as covariates at the individual-patient level and practice size as a covariate at the practice level). A 3-level mixed-model (linear- or generalised- as appropriate to numerical and categorical outcome data, respectively) will be fitted to test for the effect of the MOAC intervention from baseline across follow-up, taking into account clustering by practice (level 3) and patient (level 2) and repeat follow-up measures (level 1). P-values and 95% Confidence Intervals will be provided with estimates of effect size in the analysis of follow up data. The mixed model assumes that missing data is at least missing at random (MAR). We will examine effect estimates in relation to the indicated clinical marker of 0.3 of an effect size.

Complier Average Causal Effect Analysis (CACE)

A CACE analysis will be performed to provide an unbiased estimate of treatment effect for patients treated as per protocol specification (treatment administered as per protocol in the intervention arm is based on participants having seen the practice nurse MOAC 2 in the intervention practices).

Key secondary outcomes will be analysed including the Arthritis Self-Efficacy pain subscale [41] and the OMERACT/OARSI responder criteria [42], which combines measures of pain intensity (0 to 10 NRS) and function (subscale of the WOMAC) with global assessment of change to determine if participants are 'responders' to treatment.

Sub group analyses will be performed on the primary outcome (SF-12 PCS) as well as Arthritis Self-Efficacy pain sub-scale according to the following:

1. Age group (45 to 64 vs 65 and above);
2. Sex (male vs female);
3. Baseline SF12 – physical component score (cut off at median score);
4. Multi-site pain (less than 2 vs ≥2).

An interaction term (product of the subgroup variable and study group) will be included as an additional term in the regression models to evaluate the subgroup effect.

Sensitivity analyses will be conducted (this will be carried out on primary outcome [SF-12 PCS]), details of which can be found in Additional file 5.

Analysis of medical record data

We will split the medical records into three time periods: the 12 months before the template installation, the 6 months after installation but prior to randomisation, and the 12 months after start of the intervention. Eligible patients with OA or joint pain consultations in each time period (aged 45 years and over) will be identified. To assess the effect of the template, we will determine changes in routine recording of OA management pre- and post-template installation but prior to randomisation. This will include assessment of prescribing behaviours (paracetamol, topical NSAIDs, opioid, oral NSAIDs with or without a PPI, weight loss agents), investigation (use of relevant X-rays), and referral to selected specialities including exercise referral or physiotherapy, occupational therapy, weight loss programmes, orthopaedics, pain management, and rheumatology. We will determine the level of use of the template and the proportion of patients who have evidence of achievement of each Quality Indicator as recorded in the template in the first six months after installation. We will assess socio-demographic and clinical factors associated with achievement. Differences between the two clusters (template v. template plus intervention) on practice level outcomes will be assessed for the 12 months after the start of the intervention. The analysis will compare (i) between clusters on each of the Quality Indicator outcome measures adjusting for baseline achievement, and (ii) within clusters on change on each of the Quality Indicators from the six months pre-randomisation using multilevel modelling to adjust for clustering of patients within practices.

Analysis of HCP behaviour

General characteristics and baseline views of all GPs and nurses involved in the study will be determined. We will evaluate whether the knowledge, attitudes and beliefs, and reported practice of GPs and nurses from the intervention practices are (a) similar to the GPs and nurses from the control practices and (b) change as a result of the study training programme and involvement with OA patients over the time course of the study.

The video-recorded simulated patient consultations will be rated for the presence or absence of a number

of consultation behaviours (items), *e.g.*, did the GP elicit the patient's ideas or concerns about what the patient thinks is the matter, or did the GP tell the patient that the problem is due to OA? The overall rating for each GP for each consultation (the number of items rated as present) and the overall rating for each item at each time point (the number of GPs rated as having demonstrated that item) will be determined. The first value is a measure of the competency of a GP in delivering the model OA consultation (GP competency), and the second is a measure of the competency of all the GPs in delivering one element of the model OA consultation (item competency).

The following analyses will be undertaken:

1. The number of GPs who have increased GP competency at one month and six months post training.
2. The change in mean GP competency at one month and six months post training.
3. The change in item competency at one month and six months post training.

Analysis of qualitative data

Data will be entered into NVIVO 9 software to aid analysis. The constant comparative method [50] will be the primary analytical tool, but supplemented where appropriate by narrative analysis. The use of different data sources and methods allows for triangulation, and the development of the coding scheme. The qualitative team will carry out joint data analysis and interpretation of the Normalisation Process Theory (NPT) in relation to the data collected. The NPT will form the theoretical framework to order emerging themes and concepts across the intervention practices studied. The model focuses on the work that is required to get a new intervention integrated and workable. The key elements are: (i) the examination of how people as individuals and as a group make certain practices a reality; (2) what mechanisms promote or inhibit new practices; (3) the way in which practices are (re)produced by continuous investment by key HCPs so that it becomes part of clinical routines. By using the NPT, the new supported self-management strategy (the MOAC intervention and guidebook) will be studied in its totality, so that the findings can be more robust in terms of answering the question: does the new intervention work, under what circumstance, for whom and why?

Data analysis will take place in several stages. All interviews will then be coded, and coding schemes will be revised according to ongoing data analysis. Coded data will then be compared and emerging themes discussed. The quantitative findings will be embedded within the reporting of the analysis of the key quantitative results of the study.

Health economics analysis

The full Health Economics analysis plan is reported in Additional file 6.

Discussion

Despite the publication of National and International treatment recommendations, evidence suggests that there is a gap between the recommendations and what patients actually receive in the UK [11]. The NICE guidance (2008) highlights the possible therapeutic gains of positive self-management in primary care; however, there is as yet no evidence regarding the feasibility of implementing NICE core OA recommendations in primary care and the effect of this package on the course and impact of the condition [13]. To our knowledge, the MOSAICS study is the first to develop and evaluate a system for delivering these core messages in UK primary care.

Complex interventions are frequently employed in the NHS. Trials of complex interventions are of increasing importance because of the drive to provide the most cost effective healthcare; however, there are issues in describing, developing, documenting and reproducing complex interventions [23]. While such trials can pose a considerable challenge for researchers, approaches that incorporate both qualitative and quantitative evidence should lead to improved study design, implementation, evaluation and generalisability of results.

The MOSAICS study is a complex intervention and will use a cluster RCT design to test a novel intervention designed to increase the uptake of the core NICE OA guidelines. The primary aim is to evaluate a new model of supported self-management for OA, and evaluate the impact of this on practice level and HCP outcomes and in patients consulting with joint pain. Secondary aims are to: develop a training package for GPs and practice nurses; test the feasibility of recording the management of OA in consultations using 'quality markers' collected via a new consultation template; and describe the uptake of core NICE OA recommendations.

In line with the recommendations of the Medical Research Council (MRC) [51], this study includes a process evaluation component, which aims to explain any differences between what is expected and what actually happens in practice; and a health economic evaluation component, which will make the results of the evaluation more valuable to decision-makers.

Trial monitoring

The Research Centre's independent Data Monitoring Committee (DMC) will monitor the trial, and reports will be written in line with Arthritis Research UK recommendations (www.arthritisresearchuk.org). The trial will also be monitored by an independent trial steering committee (TSC) annually. This committee is made up of individuals

with expertise in musculoskeletal medicine, community rheumatology, biostatistics, nursing, community-based research, and health economics. The committee also includes a representative from Arthritis Care and an OA research user group. Both committees will be notified of any serious adverse events that may occur during the trial.

Patient and public involvement (PPI)

The Arthritis Research UK Primary Care Centre at Keele is committed to taking an explicit and systematic approach to involving patients and the public in research. For the MOSAICs study, the Research Users' group will work in collaboration with researchers on a wide range of tasks. These tasks will focus on aspects of research design, management and dissemination. Some examples of involvement include: development and design of the OA guidebook, advice on the content of the population survey, development of Quality Indicators for general practice consultations, involvement in developing training for HCPs and Steering Committee Membership.

Trial sponsor: Keele University

The sponsor will have no role in the design and analysis of the data.

Additional files

Additional file 1: Resources provided.
Additional file 2: Consent form.
Additional file 3: The MOAC intervention.
Additional file 4: List of Read codes.
Additional file 5: Sensitivity analyses.
Additional file 6: Health economics analysis plan.

Competing interests

The authors declare that they have no competing interest.

Authors' contribution

All authors participated in the design of the study and drafting the manuscript. All authors read and approved the final manuscript.

Authors' information

Chief Investigator: Professor Krysia Dziedzic.
Population survey: Principal Investigators: Professor Krysia Dziedzic and Dr. Martyn Lewis Consultation data and template: Principal Investigators: Dr. John Edwards and Dr. Kelvin Jordan.
Intervention & evaluation of training:
Principal Investigator (MOAC 1): Dr. Mark Porcheret.
Principal Investigators (MOAC 2): Dr. Emma Healey and Professor Chris Main.
Principal Investigator (MOAC 3): Professor Krysia Dziedzic.
Qualitative evaluation:
Principal Investigators: Dr Clare Jinks and Professor Pauline Ong.
Trial coordinator: Kris Clarkson.
Trial Statistician: Dr. Martyn Lewis with support from Ebenezer Afolabi.
Health Economist: Dr. Sue Jowett with support from Raymond Oppong.
User involvement: Dr. Clare Jinks.

Acknowledgements

This paper presents independent research commissioned by the National Institute for Health Research (NIHR) Programme Grant (RP-PG-0407-10386). The views expressed in this paper are those of the author(s) and not necessarily those of the NHS, the NIHR or the Department of Health. This research was also funded by the Arthritis Research UK Centre in Primary Care grant (Grant Number 18139). EH and CJ are part-funded by the National Institute for Health Research (NIHR) Collaborations for Leadership in Applied Health Research and Care West Midlands. JJE is supported by an In-Practice Fellowship from the National Institute for Health Research (NIHR) and AF is supported by an NIHR Doctoral, Clinical Academic Training Fellowship.
The authors would like to thank the OA Research Users' Group and the network, nursing, health informatics and administrative staff at Keele University's Arthritis Research UK Primary Care Centre for all their support and assistance with this study. The authors would like to give special thanks to all of the staff at the participating general practices and the GP facilitators Dr. Marvyn Ryles, Dr. Tayo Shoran and Dr. Beth Hanson, who provided support to the general practices involved in the study. Thanks go to Professor Robbie Foy, Professor Martin Eccles, Professor Susan Michie, Janet Grime and Vince Cooper for their valued thoughts and suggestions on the developmental work for this trial. The authors would also like to thank all members of the Trial Steering Committee and Data Monitoring Committee for their valuable input in study design and conduct.

Author details

[1]Arthritis Research UK Primary Care Centre, Keele University, Keele, Staffordshire ST5 5BG, UK. [2]The School of Nursing, Midwifery and Social Work, The University of Manchester, Manchester M13 9PL, UK. [3]The Haywood Hospital, Staffordshire Rheumatology Centre, High Lane, Burslem, Stoke-On-Trent, Staffordshire ST6 7AG, UK. [4]School of Health and Population Sciences, College of Medical and Dental Sciences, University of Birmingham, Edgbaston, Birmingham B15 2TT, UK. [5]Nottingham Clinical Trials Unit, Nottingham Health Science Partners, C Floor, South Block, Queen's Medical Centre, Nottingham NG7 2UH, UK.

Received: 17 April 2014 Accepted: 8 July 2014
Published online: 27 August 2014

References

1. Jinks C, Ong BN, Richardson J: **A mixed methods study to investigate needs assessment for knee pain and disability: population and individual perspectives.** *BMC Musculoskelet Disord* 2007, **8**:59.
2. Hill S, Dziedzic K, Thomas E, Baker SR, Croft P: **The illness perceptions associated with health and behavioural outcomes in people with musculoskeletal hand problems: findings from the North Staffordshire OA Project (NorStOP).** *Rheumatology* 2007, **46**:944–951.
3. Altman RD, Hochberg MC, Moskowitz RW, Schnitzer TJ: **Recommendations for the medical management of OA of the hip and knee: 2000 update.** *Arthritis Rheum* 2000, **43**:1905–1915.
4. Jordan KM, Arden NK, Doherty M, Bannwarth B, Bijlsma JWJ, Dieppe P, Gunther K, Hauselmann H, Herrero-Beaumont G, Kaklamanis P, Lohmander S, Leeb B, Lequesne M, Mazieres B, Martin-Mola E, Pavelka K, Pendleton A, Punzi L, Serni U, Swoboda B, Verbruggen G, Zimmerman-Gorska I, Dougados M, Standing Committee for International Clinical Studies Including Therapeutic Trials ESCISIT: **EULAR Recommendations 2003: an evidence based approach to the management of knee OA: Report of a Task Force of the Standing Committee for International Clinical Studies Including Therapeutic Trials (ESCISIT).** *Ann Rheum Dis* 2003, **62**:1145–1155.
5. Zhang W, Moskowitz RW, Nuki G, Abramson S, Altman RD, Arden N, Bierma-Zeinstra S, Brandt KD, Croft P, Doherty M, Dougados MD, Hochberg M, Hunter DJ, Kwoh K, Lohmander LS, Tugwell P: **OARSI recommendations for the management of hip and knee OA, Part I: Critical appraisal of existing treatment guidelines and systematic review of current research evidence.** *OA Cartilage* 2007, **15**:981–1000.
6. Zhang W, Doherty M, Leeb BF, Arden N, Bijlsma J, Dincer F, Dziedzic K, Häuselmann HJ, Herrero-Beaumont G, Kaklamanis P, Lohmander S, Maheu E, Martín-Mola E, Pavelka K, Punzi L, Reiter S, Sautner J, Smolen J, Verbruggen G, Zimmermann-Górska I: **EULAR evidence based recommendations for the management of hand OA - report of a task force of the EULAR Standing Committee for International Clinical Studies Including Therapeutics (ESCISIT).** *Ann Rheum Dis* 2007, **66**:377–388.
7. Zhang W, Moskowitz RW, Nuki G, Abramson S, Altman RD, Arden N, Bierma-Zeinstra S, Brandt KD, Croft P, Doherty M, Dougados M, Hochberg M, Hunter DJ, Kwoh K, Lohmander LS, Tugwell P: **OARSI recommendations for the**

management of hip and knee OA, Part II: OARSI evidence-based, expert consensus guidelines. *OA Cartilage* 2008, **16:**137–162.
8. Zhang W, Doherty M, Leeb BF, Alekseeva L, Arden N, Bijlsma J, Dincer F, Dziedzic K, Hauselmann HJ, Kaklamanis P, Kloppenburg M, Lohmander LS, Maheu E, Martin-Mola E, Pavelka K, Punzi L, Reiter S, Smolen J, Verbruggen G, Watt I, Zimmermann-Gorska I: **EULAR evidence based recommendations for the diagnosis of hand OA - report of a task force of the EULAR Standing Committee for International Clinical Studies Including Therapeutics (ESCISIT).** *Ann Rheum Dis* 2009, **68:**8–17.
9. Zhang W, Doherty M, Peat G, Bierma-Zeinstra MA, Arden NK, Bresnihan B, Herrero-Beaumont G, Kirschner S, Leeb BF, Lohmander LS, Mazières B, Pavelka K, Punzi L, So AK, Tuncer T, Watt I, Bijlsma JW: **EULAR evidence based recommendations for the diagnosis of knee OA.** *Ann Rheum Dis* 2010, **69:**483–489. doi:10.1136/ard.2009.113100.
10. Conaghan PG, Dickson J, Grant RL: **Care and management of OA in adults: summary of NICE guidance.** *BMJ* 2008, **336:**502–503.
11. Porcheret M, Jordan K, Jinks C, with the Primary Care Rheumatology Society: **Primary care treatment of knee pain a survey in older adults.** *Rheumatol* 2007, **46:**1694–1700.
12. Steel N, Bachmann M, Maisey S, Shekelle P, Breeze E, Marmot M, Melzer D: **Self reported receipt of care consistent with 32 Quality Indicators: national population survey of adults aged 50 or more in England.** *BMJ* 2008, **337:**a957.
13. NICE: **OA: The care and management of adults with OA.** In *National Institute of Health and Clinical Excellence.* 2008. http://www.nice.org.uk/guidance/CG59.
14. Finney A, Porcheret M, Grime J, Jordan KP, Handy J, Healey EL, Ryan S, Jester R, Dziedzic KS: **Defining the content of an opportunistic osteoarthritis consultation with primary health care professionals: A dephi consensus study.** *Arthritis Care Res* 2013, **65:**962–8. doi:10.1002/acr.21917.
15. Porcheret M, Grime J, Main C, Dziedzic K: **Developing a model osteoarthritis consultation: a Delphi consensus exercise.** *BMC Musculoskelet Disord* 2013, **14:**25.
16. Department of Health: *The NHS improvement plan: putting people at the heart of public services.* London: Department of Health; 2004.
17. Mann C, Gooberman-Hill R: **Health Care Provision for Osteoarthritis: Concordance Between What Patients Would Like and What Health Professionals Think They Should Have.** *Arthritis Care Res* 2011, **63:**963–972. doi:10.1002/acr.20459.
18. Kralik D, Koch T, Price K, Howard N: **Chronic illness self-management: taking action to create order.** *J Clin Nurs* 2004, **13:**259–267.
19. Thorne S, Paterson BL, Russell C: **The structure of everyday self-care decision making.** *Qual Health Res* 2003, **13:**1337–1352.
20. Townsend A, Wyke S, Hunt K: **Self-managing and managing self: practical and moral dilemmas in accounts of living with chronic illness.** *Chronic Illn* 2006, **2:**185–195.
21. Kendall E, Rogers A: **Extinguishing the social?: state sponsored self-care policy and the Chronic Disease Self-management Programme.** *Disability Soc* 2007, **22:**129–143.
22. Taylor D, Bury M: **Chronic Illness, expert patients and care transition.** *Sociol Health Illness* 2007, **29:**27–45.
23. Campbell M, Fitzpatrick R, Haines A, Kinmonth AL, Sandercock P, Spiegelhalter D, Tyrer P: **Framework for design and evaluation of complex interventions to improve health.** *BMJ* 2010, **340:**c869. doi:10.1136/bmj.c869.
24. Kennedy AP, Rogers AE, Bower P: **Support for self care for patients with chronic disease.** *BMJ* 2007, **335:**968–970.
25. Kennedy AP, Nelson E, Reeves D, Richardson G, Roberts C, Robinson A, Rogers AE, Sculpher M, Thompson DG, the North-West Regional Gastrointestinal Research Group: **A randomised controlled trial to assess the effectiveness and cost of a patient orientated self-management approach to chronic inflammatory bowel disease.** *Gut* 2004, **53:**1639–1645.
26. Robinson A, Lee V, Kennedy A, Middleton L, Rogers A, Thompson DG, Reeves D: **A randomised controlled trial of self-help interventions in patients with a primary care diagnosis of irritable bowel syndrome.** *Gut* 2006, **55:**643–648.
27. Kennedy A, Bower P, Reeves D, Blakeman T, Bowen R, Chew-Graham C, Eden M, Fullwood C, Gaffney H, Gardner C, Lee V, Morris R, Protheroe J, Richardson G, Sanders C, Swallow A, Thompson D, Rogers A: **Implementation of self management support for long term conditions in routine primary care settings: cluster randomised controlled trial.** *BMJ* 2013, **346:**f2882. doi:http://dx.doi.org/10.1136/bmj.f2882.
28. Michie S, Johnston M, Abraham C, Lawton R, Parker D, Walker A: **Making psychological theory useful for implementing evidence based practice: a consensus approach.** *Qual Saf Health Care* 2005, **14:**26–33. doi:10.1136/qshc.2004.011155.
29. Michie S, Johnston M, Francis J, Hardeman W, Eccles M: **From theory to Intervention: Mapping Theoretically Derived Behavioural Determinants to Behaviour Change Techniques.** *Applied Psychol* 2008, **57:**66–680.
30. Michie S, van Stralen MM, West R: **The behaviour change wheel: A new method for characterising and designing behaviour change interventions.** *Implementation Sci* 2011, **6:**42. doi:10.1186/1748-5908-6-42.
31. Grol R: **Beliefs and evidence in changing clinical practice.** *BMJ* 1997, **315:**418–421.
32. Silverman J, Kurt S, Draper J: *Skills for Communicating with Patients.* 2nd edition. Oxford: Radcliffe Publishing; 2005.
33. May T: *Social Research: Issues, methods and process.* Buckingham: Open University Press; 1993.
34. May C, Finch T: **Implementing, embedding and integrating practices: an outline of Normalisation Process Theory.** *Sociol* 2009, **43:**535–554.
35. Chan A-W, Tetzlaff JM, Altman DG, Laupacis A, Gøtzsche PC, Krleža-Jerić K, Hróbjartsson A, Mann H, Dickersin K, Berlin J, Doré C, Parulekar W, Summerskill W, Groves T, Schulz K, Sox H, Rockhold FW, Rennie D, Moher D: **SPIRIT: Statement: Defining standard protocol items for clinical trials.** *Ann Intern Med* 2013, **2013**(158):200–207.
36. Chan A-W, Tetzlaff JM, Gøtzsche PC, Altman DG, Mann H, Berlin J, Dickersin K, Hróbjartsson A, Schulz KF, Parulekar WR, Krleža-Jerić K, Laupacis A, Moher D: **SPIRIT 2013b Explanation and Elaboration: Guidance for protocols of clinical trials.** *BMJ* 2013, **346:**e7586.
37. Dziedzic KS, Healey EL, Main CJ: **Implementing the NICE osteoarthritis guidelines in primary care: a role for practice nurses.** *Musculoskeletal Care* 2013, **11:**1. doi:10.1002/msc.1040.
38. Grime J, Dudley B: **Developing written information on OA for patients: facilitating user involvement by exposure to qualitative research.** *Health Expect* 2011. doi:10.1111/j.1369-7625.2011.00741.
39. Norman GR, Sloan JA, Wyrwich KW: **Interpretation of changes in health-related quality of life: the remarkable universality of half a standard deviation.** *Med Care* 2003, **41:**582–92.
40. Ware JE, Kosinski M, Keller SD: **A 12-item short form health survey (SF-12): Construction and preliminary tests of reliability and validity.** *Med Care* 1996, **32:**220–33.
41. Lorig K, Chastain R, Ung E, Shoor S, Holman HR: **Development and evaluation of a scale to measure perceived self-efficacy in people with arthritis.** *Arthritis Rheum* 1989, **32:**37–44.
42. Pham T, van der Heijde D, Altman RD, Anderson JJ, Bellamy N, Hochberg M, Simon L, Strand V, Woodworth T, Dougados M: **OMERACT-OARSI initiative: Osteoarthritis Research Society International set of responder criteria for osteoarthritis clinical trials revisited.** *Osteoarthritis Cartilage* 2004, **12:**389–399.
43. Angrist JD, Imbens GW, Rubin DB: **Identification of causal effects using instrumental variables.** *J Am Stat Assoc* 1996, **91:**444–455.
44. Spitzer RL, Kroenke K, Williams JB, Löwe B: **A brief measure for assessing generalized anxiety disorder: the GAD-7.** *Arch Intern Med* 2006, **166:**1092–1097.
45. Group EQ: **EuroQol: a new facility for the measurement of health-related quality of life.** *Health Policy* 1990, **16:**199–208.
46. Matthews CE, Ainsworth BE, Hanby C, Pate RR, Addy C, Freedson PS, Jones DA, Macera CA: **Development and testing of a short physical activity recall questionnaire.** *Med Sci Sports Exerc* 2005, **37:**986–994.
47. Jinks C, Jordan K, Ong BN, Croft P: **A brief screening tool for knee pain in primary care (KNEST) 2. Results from a survey in the general population aged 50 and over.** *Rheumatology* 2004, **43:**55–61.
48. Edwards JJ, Khanna M, Jordan KP, Jordan JL, Bedson J, Dziedzic KS: **Quality indicators for the primary care of osteoarthritis: a systematic review.** *Ann Rheum Dis* 2013. Published On-line First: 27 November 2013. doi:10.1136/annrheumdis-2013-203913.
49. Mead N, Bower P, Roland M: **The General Practice Assessment Questionnaire (GPAQ) – Development and psychometric characteristics.** *BMC Fam Pract* 2008, **9:**13. doi:10.1186/1471-2296-9-13.
50. Charmaz K: *Constructing grounded theory: A Practical Guide through Qualitative Analysis.* London: Sage Publications Ltd; 2006.
51. Craig P, Dieppe P, Macintyre S, Michie S, Nazareth I, Petticrew M: **Developing and evaluating Complex interventions: new guidance.** *MRC* 2008. http://www.mrc.ac.uk/documents/pdf/complex-interventions-guidance/.

52. Bellamy N, Buchanan WW, Goldsmith CH, Campbell J, Stitt LW: **Validation study of WOMAC: a health status instrument for measuring clinically important patient relevant outcomes to antirheumatic drug therapy in patients with osteoarthritis of the hip or knee.** *J Rheumatol* 1988, **15:**1833–1840.
53. Meenan RF, Mason JH, Anderson JJ, Guccione AA, Kazis LE: **AIMS2: The content and properties of a revised and expanded Arthritis Impact Measurement Scales Health Status Questionnaire.** *Arthritis Rheum* 1992, **35:**1–10.
54. Craig CL, Marshall AL, Sjöström M, Bauman AE, Booth ML, Ainsworth BE, Pratt M, Ekelund U, Yngve A, Sallis JF, Oja P: **International physical activity questionnaire: 12-country reliability and validity.** *Med Sci Sports Exerc* 2003, **35:**1381–1395.
55. Washburn RA, Smith K, Jette AM, Janney CA: **The physical activity scale for the elderly (PASE): development and evaluation.** *J Clin Epi* 1993, **46:**153–162.
56. van der Windt DA, van der Heijden GJ, de Winter AF, Koes BW, Deville W, Bouter L: **The responsiveness of the Shoulder Disability Questionnaire.** *Ann Rheum Dis* 1998, **57:**82–87.
57. Kroenke K, Strine TW, Spitzer RL, Williams JBW, Berry JT, Mokdad AH: **The PHQ-8 as a measure of current depression in the general population.** *J Affective Disorders* 2009, **114:**163–173.
58. Brazier JE, Roberts J: **The Estimation of a Preference-Based Measure of Health from the SF-12.** *Med Care* 2004, **42:**851–859.
59. Al-Janabi H, Flynn T, Coast J: **Development of a self-report measure of capability wellbeing for adults: the ICECAP-A.** *Qual Life Res* 2012, **21:**167–176.
60. Østerås N, Garratt A, Grotle M, Natvig, Kjeken I, Kvien TK, Hagen KB: **Patient-reported quality of care for osteoarthritis: development and testing of the osteoarthritis quality indicator questionnaire.** *Arthritis Care Res* 2013, **65:**1043–1051.
61. Howie J, Heaney DJ, Maxwell M, Walker JJ: **A comparison of a Patient Enablement Instrument (PEI) against two established satisfaction scales as an outcome measure of primary care consultations.** *Fam Pract* 1998, **15:**165–171.

doi:10.1186/s13012-014-0095-y
Cite this article as: Dziedzic *et al.*: Implementing the NICE osteoarthritis guidelines: a mixed methods study and cluster randomised trial of a model osteoarthritis consultation in primary care - the Management of OsteoArthritis In Consultations (MOSAICS) study protocol. *Implementation Science* 2014 **9**:95.

Submit your next manuscript to BioMed Central and take full advantage of:

- **Convenient online submission**
- **Thorough peer review**
- **No space constraints or color figure charges**
- **Immediate publication on acceptance**
- **Inclusion in PubMed, CAS, Scopus and Google Scholar**
- **Research which is freely available for redistribution**

Submit your manuscript at
www.biomedcentral.com/submit

BioMed Central
